# CoAIMs: A Cost-Effective Panel of Ancestry Informative Markers for Determining Continental Origins

**DOI:** 10.1371/journal.pone.0013443

**Published:** 2010-10-15

**Authors:** Eric R. Londin, Margaret A. Keller, Cathleen Maista, Gretchen Smith, Laura A. Mamounas, Ran Zhang, Steven J. Madore, Katrina Gwinn, Roderick A. Corriveau

**Affiliations:** 1 Coriell Institute for Medical Research, Camden, New Jersey, United States of America; 2 National Institute for Neurological Disorders and Stroke, Bethesda, Maryland, United States of America; University of Utah, United States of America

## Abstract

**Background:**

Genetic ancestry is known to impact outcomes of genotype-phenotype studies that are designed to identify risk for common diseases in human populations. Failure to control for population stratification due to genetic ancestry can significantly confound results of disease association studies. Moreover, ancestry is a critical factor in assessing lifetime risk of disease, and can play an important role in optimizing treatment. As modern medicine moves towards using personal genetic information for clinical applications, it is important to determine genetic ancestry in an accurate, cost-effective and efficient manner. Self-identified race is a common method used to track and control for population stratification; however, social constructs of race are not necessarily informative for genetic applications. The use of ancestry informative markers (AIMs) is a more accurate method for determining genetic ancestry for the purposes of population stratification.

**Methodology/Principal Findings:**

Here we introduce a novel panel of 36 microsatellite (MSAT) AIMs that determines continental admixture proportions. This panel, which we have named Continental Ancestry Informative Markers or CoAIMs, consists of MSAT AIMs that were chosen based upon their measure of genetic variance (F_st_), allele frequencies and their suitability for efficient genotyping. Genotype analysis using CoAIMs along with a Bayesian clustering method (STRUCTURE) is able to discern continental origins including Europe/Middle East (Caucasians), East Asia, Africa, Native America, and Oceania. In addition to determining continental ancestry for individuals without significant admixture, we applied CoAIMs to ascertain admixture proportions of individuals of self declared race.

**Conclusion/Significance:**

CoAIMs can be used to efficiently and effectively determine continental admixture proportions in a sample set. The CoAIMs panel is a valuable resource for genetic researchers performing case-control genetic association studies, as it can control for the confounding effects of population stratification. The MSAT-based approach used here has potential for broad applicability as a cost effective tool toward determining admixture proportions.

## Introduction

Population stratification refers to the subdivision of a population into different ancestral groups having different allele frequencies and different disease prevalence. In contemporary populations there has been recent admixture between individuals from different populations resulting in variable ancestry, e.g. in African American and Hispanic populations. Population stratification can act as a confounding factor in genetic studies, such as genome-wide association studies (GWAS), where the presence of uncontrolled population structure can lead to false-positive or false-negative findings [1]–[3]. Moreover, to date the majority of GWAS have used relatively homogeneous sample sets, generally made up of individuals of European decent [4]. Thus, it is not clear if the results of many of these studies can be generalized to other populations. As additional studies are conducted in multiethnic populations the adverse effects of population stratification are likely to arise [5], [6]. Additionally, different population groups show disparities in disease prevalence, morbidity rates, and treatment response [7]. For example, in breast cancer [8] and diabetes incidence, prevalence, and severity [9] are known to vary across different ancestral populations. Similarly, genetic ancestry can be used to predict response to standard hepatitis C treatment [10]–[12]. These and similar findings demonstrate that ethnicity can be a risk factor for the development of disease and a predictor of responses to treatment. In this regard, addressing population stratification is of increasing relevance given the development of personalized medicine.

Reliably detecting population structure can be difficult. Perhaps the simplest approach to control for population stratification is to use the self-reported race/ethnicity of the study participants. While in some populations and for some studies this information may be sufficient, it is inadequate when considerable admixture is present, such as exists in North America [5], [13], [14]. Even though individuals might self-identify with a single racial or ethnic category, recent studies have shown that this information is often incorrect due to the presence of admixture. For example, Hispanicity refers to a diverse range of people of Cuban, Mexican, Puerto Rican, South or Central American, or of other Spanish culture or origin, regardless of race. In fact, previous studies of Hispanic populations demonstrated a trihybrid ancestral population structure consisting of Caucasian, Native American and African populations, with the proportions of these ancestral population groups varying greatly [15]. Moreover, for multiethnic groups, self-declared ancestry is not useful for the purposes of genetic characterization [5], [13], [14].

An alternative and more accurate approach to detect population stratification is to determine genetic ancestry using AIMs. AIMs are polymorphic markers that exhibit high allele frequency differences among parental populations (e.g., African vs. European) and can be used to accurately estimate individual admixture and identify population structure [16]–[18]. The two types of AIMs commonly used are single nucleotide polymorphisms (SNPs) and short tandem repeat polymorphisms (STRs or microsatellites, MSATs) [13], [16], [19]–[22]. While previous studies have used many hundreds to thousands of these markers to determine population genetic structure [23]–[25], genotyping this number of markers in many samples is not feasible for many laboratories due to cost and time considerations. Therefore, there is a need to ascertain the same information using a small number of markers. Using highly informative AIMs can reduce the number of markers required, which in turn reduces the time and cost necessary to obtain accurate ancestral information.

We have developed CoAIMs, a comprehensive panel of 36 MSATs suitable for contemporary genetic research with the potential future clinical applications. This set of AIMs (1) differentiates among continental groups including Europe/Middle East (Caucasians), East Asia, Africa, Native America, and Oceania, (2) accurately measures individual ancestry proportions in admixed populations, and (3) is efficient and cost-effective.

## Materials and Methods

### Population Controls and Self-Declared Ancestry Samples

DNA samples used as reference material for parental populations were generously provided by the National Institute of General Medical Sciences (NIGMS) and the National Human Genome Research Institute (NHGRI). These include 234 samples from the NIGMS Human Population Collection as follows: Oceania (5 Melanesian, 7 Pacific Islander); East Asia (10 Taiwan Ami, 10 Taiwan Atayal); Native American, also referred to as Americas (5 Brazil Karitiana, 4 Mayan, 5 Pima, 5 Quechua, 10 South American Andes, 4 Suri and 20 other including from Brazil, Guyana Mexico and Venezuela); Africans of sub-Saharan ancestry (5 Mbuti, 5 Biaka, 16 of unspecified group); Caucasian, Europe (10 Czechoslovakian, 8 Greek, 9 Hungarian, 10 Iberian, 11 Icelandic, 10 Basque, 9 Krasnodar from Southeast Russia, 10 Zversky from Northeast Russia, 10 Northern European unspecified); Caucasian, Middle East (5 Druze, 11 Ashkenazi Jewish, 10 Iranian Jewish, and 10 Moroccan Jewish). The population controls also included 476 HapMap samples from NHGRI as follows: East Asia (45 Japanese, 45 Han Chinese); Africans of sub-Saharan ancestry (90 Luhya, 90 Yoruba); Caucasian, Europe (116 CEPH, 90 Tuscan). 385 samples from the National Institute for Neurological Disorders and Stroke (NINDS) Repository generously provided 385 samples with self-declared ancestry: 92 Caucasian from North America (NDPT020), 92 African American (NDPT111), 20 Asian American, 92 Caucasian Hispanic from North America (NDPT112), 20 non-Caucasian Hispanic from North American, 25 American Indian, and 14 Pacific Islander and 30 of undeclared or mixed race. Genomic DNA derived from either peripheral whole blood or lymphoblastoid cell lines, were utilized in this study. No human subjects were recruited for this study; de-identified samples were obtained from the NIGMS, NHGRI and NINDS Repositories at Coriell. A list of samples used can be found in **Table S1**. All samples are available from Coriell Cell Repositories at the Coriell Institute for Medical Research (Camden, NJ; http://ccr.coriell.org/).

Genotype data from the Human Genome Diversity Panel [26] were downloaded from Dr. Noah Rosenberg's database (http://rosenberglab.bioinformatics.med.umich.edu/). The dataset included genotype results for 783 MSATS on 1048 samples from 7 distinct worldwide geographical locations. This panel of samples has been used in multiple population genetic studies [24], [25], [27]. Here we used the dataset to identify a small panel of markers that can differentiate among the continental population groups.

### Genotyping

Population controls from the NIGMS and NHGRI collections were initially genotyped using the ABI Identifiler panel (Applied Biosystems, Foster City, CA) comprised of the 15 Combined DNA Index System (CODIS) markers, and the Coriell Identity Mapping kit (6-plex) (http://ccr.coriell.org/Sections/Search/MSK.aspx?Ref=MSK&PgId=202) [28], per the manufacturers' recommended protocols (see **Table S2** for a list of the MSATs).

The CoAIMs panel was optimized to be genotyped in three multiplex PCR reactions, each containing 12 primer pairs (**Table S3**). The groupings were based upon MSAT base pair size ranges. One primer from each pair was fluorescently-end labeled with PET, VIC, 6FAM, and NED (Applied Biosystems, Foster City, CA). Each PCR was setup with 30 ng of genomic DNA, 0.4 µl AmpliTaq Gold (5 U/µl), 5.0 µl primer mix, 2.0 µl dNTPs (2.5 mM), 2.0 µl MgCl_2_ (25mM), 2.5 µl 10× PCR buffer, and deionized H_2_O to 25 µl and the PCR cycling conditions are in **Table S4**. The multiplex PCR products were analyzed by capillary electrophoresis using an Applied Biosystems 3730 DNA Analyzer. The NIGMS and NHGRI parental population samples were genotyped to set marker bins to generate the expected genotype calls using the ABI GeneMapper v3.5 software as well to analyze the fragment sizes, colors and intensities of the PCR products.

### Statistical Analyses

Population structure was inferred using a Bayesian clustering approach implemented using STRUCTURE v2.3 [29]–[31] software. By identifying individuals with similar allele frequencies, this program assigns individuals to populations, infers the number of parental populations (K) and estimates admixture proportions for individuals. This clustering approach estimates shared ancestry of individuals based on their genotypes and infers individual proportions of ancestry from “K” clusters, where K is specified in advance and corresponds to the hypothetical number of ancestral populations. The best fit K is evaluated using StructureSum (see below). Individuals can be assigned admixture estimates from multiple ancestral populations, with the estimates summing to 1 across the population clusters. All STRUCTURE runs were performed without any prior population assignment, and employed the admixture model with a 400,000 step burn-in and 350,000 Markov chain Monte Carlo (MCMC) iterations. All analyses were performed using the “infer α” option with a separate α estimated for each population (α characterizes the Dirichlet parameter for the degree of admixture). Runs were performed with λ = 1, where λ parameterizes prior probability of allele frequency based upon the Dirchlet distribution.. Since STRUCTURE assigns cluster assignments in each run, CLUMPP software [32] was used to combine multiple STRUCTURE runs for a particular value of K by averaging the cluster assignment values from different runs for individuals to produce average cluster membership values. These average values were used in the *Distruct* program [33] to produce graphs of STRUCTURE output.

To determine the best estimate or “fit” of the correct number of population clusters (K), all sets of markers tested were run with varying numbers of markers with K ranging from K = 2 to K = 12, and five replicates performed at each value of K. To statistically determine the correct number (K) of clusters for a given dataset, we used StructureSum, an R script [34] that employs the Evano et al 2005 method [35]. This algorithm detects the uppermost value of K that can be clearly resolved based upon the rate of change in the lnP(D) between successive K values.

Principal component analysis (PCA) was performed to test further the number of population clusters observed with STRUCTURE. This method can be used to infer population structure by clustering samples into groups based upon ancestral groups [36]. Briefly, PCA is a method that reduces the dataset into continuous axes of variation consisting of a smaller (reduced) number of dimensions than in the original dataset that describes the variability present in the full original dataset. When applied to genetic data with ancestry differences between samples, the axes of variation have a geographic interpretation. The top principal components (PCs) are continuous axes of variation that reflect the largest proportion of, in this case, genetic variation among subpopulations in a sample set. Individuals with PC values that are similar, and thus form a cluster when plotted, have similar ancestry. PCA was performed using the EIGENSTRAT statistical package, a part of HelixTree 7.0 software (Golden Helix, Bozeman, MT). The MSAT data were re-coded into a “false SNP” format by scoring the presence or absence of each allele [36].

F-statistic (F_st_) is a measure of surplus of homozygotes within subpopulations, and is also used to examine the overall genetic divergence among subpopulations. F_st_ values range from 0 to 1; markers with the highest values are most informative for ancestry determination [37]. The FSTAT Version 2.9.3 program [38], which applies the Wier and Cockerham algorithm [37], was used to calculate F_st_ values for each genetic marker tested, and was also used to perform pairwise F_st_ calculations. Pairwise F_st_ values provide a measure of the inter-population genetic variance as compared to intra-population genetic variance.

## Results

### ABI Identifiler and the Coriell Identity Mapping Kit Do Not Differentiate Continental Ancestry

**Figure 1:**
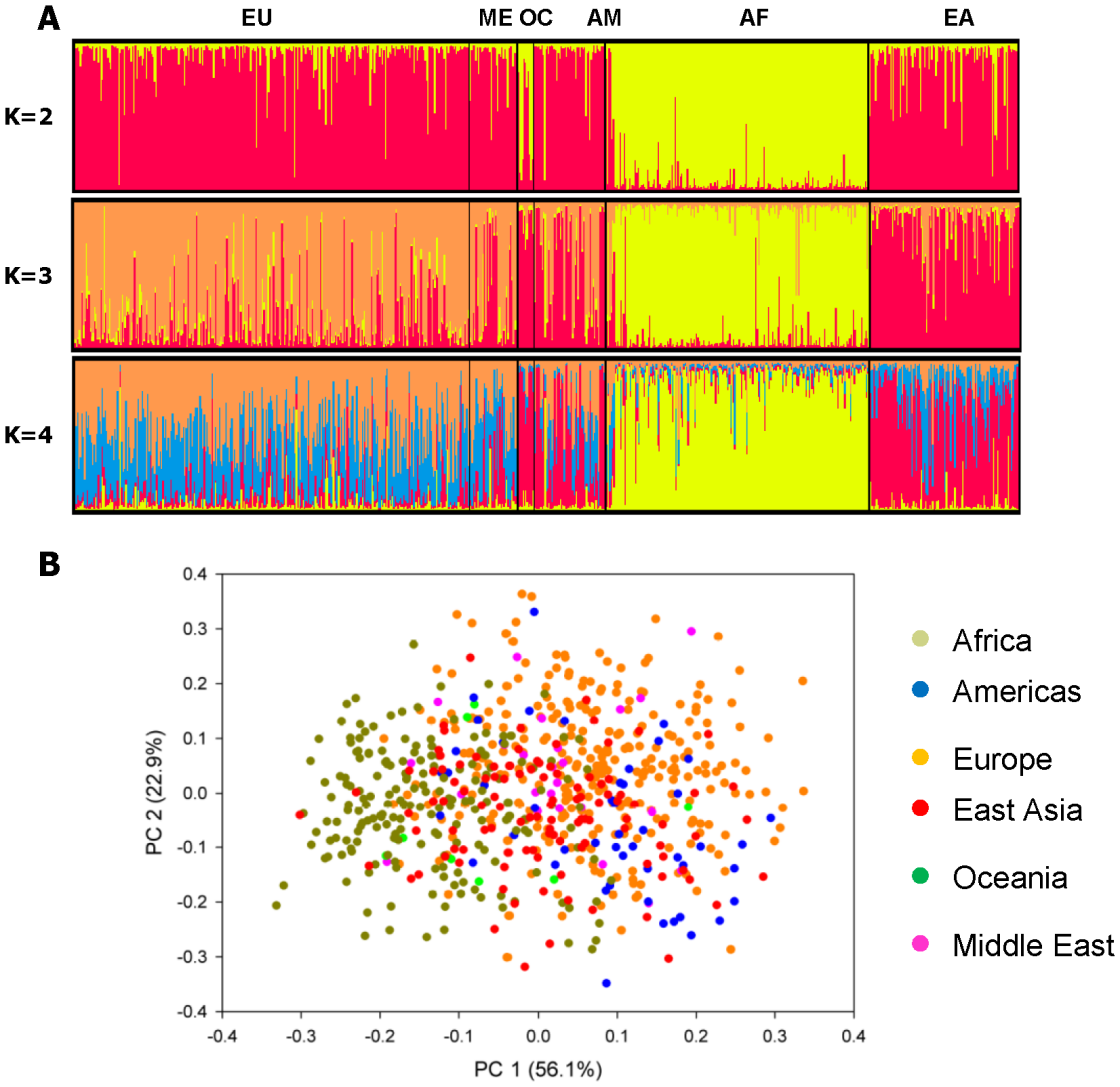
The Set of 19 MSATs (ABI Identifiler and Coriell 6-plex) Do Not Adequately Distinguish Among Continental Groups. (A) STRUCTURE results based upon 19 MSAT genotypes from 710 samples from 6 geographical regions at K = 2 through K = 4. Each individual is represented by a thin vertical line, which is portioned into K colored segments representing the individual's estimated assignment in K clusters. Black lines separate individuals into different populations (EU-Europe; ME- Middle East; OC- Oceania; AM- Americas; AF- Africa; EA- East Asia). (B) PCA results based upon the 19 markers.

We determined if combining two MSAT panels used at Coriell as part of routine quality control and using current software tools would be useful for determining population structure. The markers in both the ABI Identifiler panel and the Coriell Identity Mapping Kit (6-plex) were developed to identify unique individuals [39], [40]. The ABI Identifiler marker set is comprised of 15 MSATs, while the Coriell Identity Mapping Kit 6-plex panel consists of 6 MSATs. Two of the markers, THO-1 and VWA31, are present in both panels, yielding a combined set of 19 markers (**Table S2**). Parental population samples from 6 continental regions (**Table S2**) were genotyped using the combined marker panel and the results were analyzed using both STRUCTURE [29]–[31] and PCA [36] (**Figure 1**). Initial STRUCTURE analyses were performed under assumptions of different numbers of population groups (K) ranging from two to twelve (K = 2 to K = 12) without any pre-assignment of population affiliation. At K = 2 STRUCTURE identified one cluster of individuals of African descent and a second cluster of individuals from all other major continental population groups (**Figure 1A**: East Asia, Oceania, Native American, and Caucasian). At K = 3 individuals of Caucasian and East Asian origins begin to separate from African groups, although there is considerable noise in the data and it is not possible to assign distinct Caucasian or East Asian clusters. The addition of a fourth group (K = 4) does not improve resolution. With this set of markers, the best number of populations based upon the StructureSum algorithm is two (K = 2). The results suggest that this group of 19 markers does not adequately discern continental population structure. At best, it can distinguish between African and non-African population groups.

The genotype data generated with the 19 marker panel were analyzed using PCA. Individual specific Principal Component (PC) values, when plotted, can be interpreted according to geographic origins [36], [41], [42]. Using PCA with AIMs, individuals from different continental regions can be expected to fall into distinct and separate clusters. The top two PCs explain 77% of the variance of the data and results with populations clustering into one group (**Figure 1B**). Adding additional PCs do not further cluster samples into additional population groups (**Figure S1**). These results further that this marker panel is insufficient for determining genetic ancestry, and indicate that a more informative set of MSAT AIMs is required to adequately address genetic ancestry.

To identify an informative set of MSAT AIMs to determine genetic ancestry, a two step approach was undertaken. The first was to identify a minimal panel of markers through the *in silico* analysis of publically available genotype data. Following the identification of a set of markers, the second step is to independently confirm the ability of these markers to distinguish among population groups in a separate set of population samples. Finally, the ability of the markers to assess continental admixture proportions in samples of self-declared ancestry will be tested.

### *In Silico* Identification of a Small Set of AIMs that can Distinguish among Continental Population Groups

**Figure 2:**
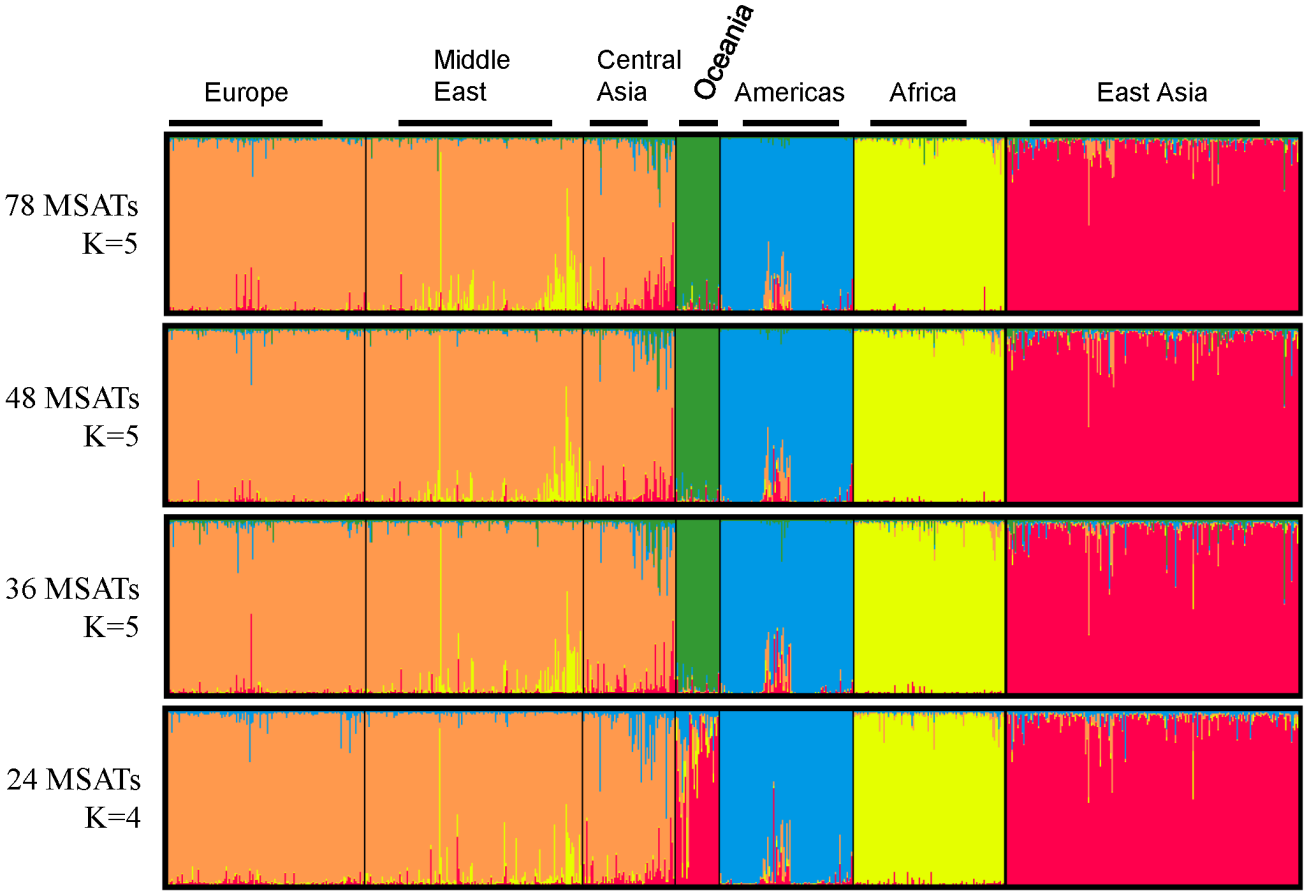
Strucuture Analysis of the HGDP Marker Sets. STRUCTURE analysis of four sets of markers consisting of 78, 48, 36 and 24 MSATs. Shown is the STRUCTURE plot with the highest probability of the number of population clusters K as determined by the SructureSum program. For the 78, 48 and 36 marker sets, plots of K = 5 is shown. Here, five continental population regions can be distinguished representing African, Americas, East Asian, Oceania and Caucasian populations. Reducing the number of markers to 24 results in four population clusters (K = 4) being distinguished (see **Table 1**).

**Figure 3:**
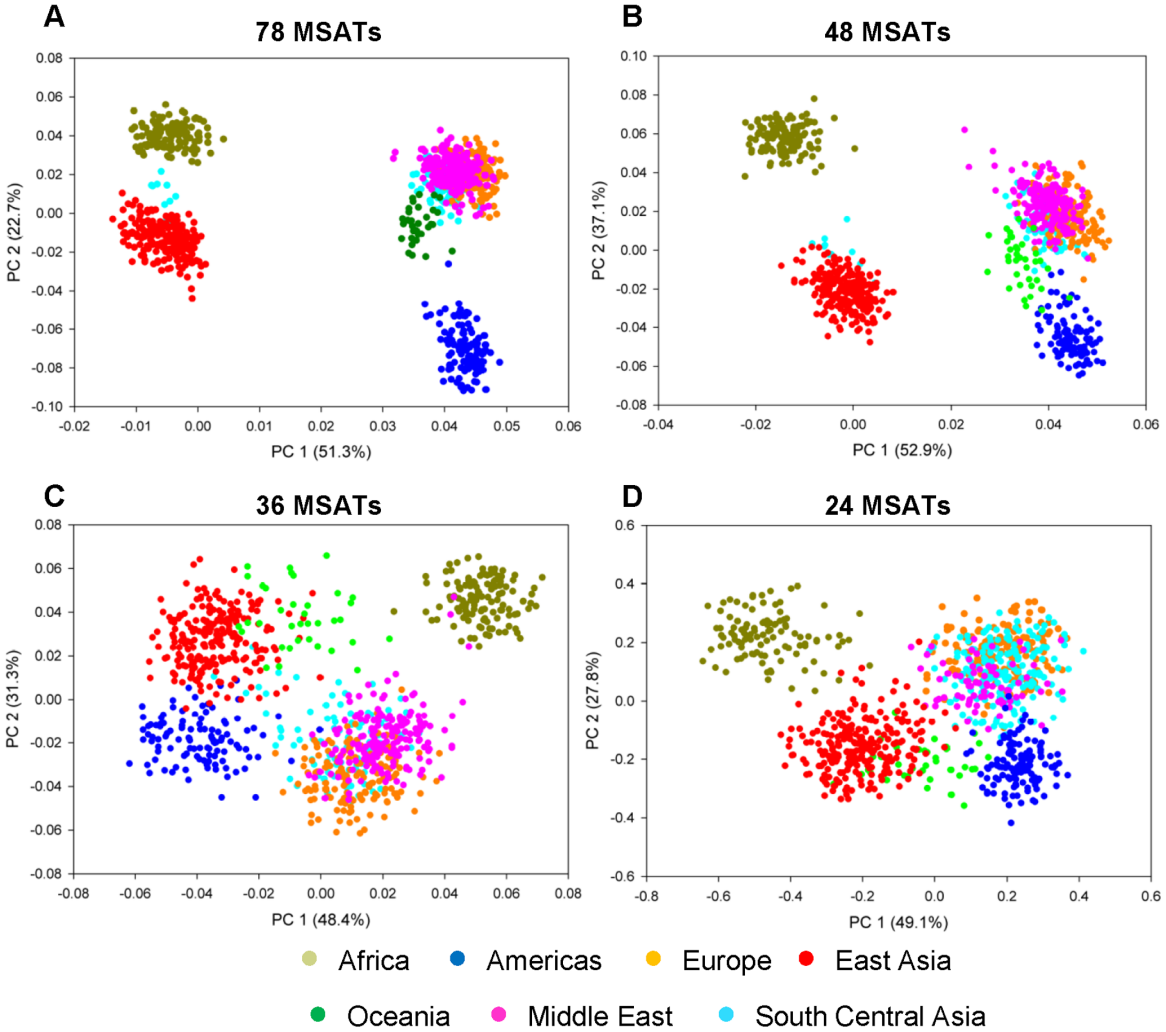
PCA of the HGDP Marker Sets. PCA plots of the (A) 78, (B) 48, (C) 36, and (D) 24 MSAT sets from the HGDP. The top two PCs are plotted. The percent of the variance explained by each component is labeled next to the axis.

*In Silico* identification of a panel of markers involved the analysis of downloaded genotype data from the HGDP (see Materials and Methods). HGDP samples have been widely used in population genetic studies to determine human population structure in fine detail [24], [27]. Accordingly, F-statistic (F_st_) values were determined for all 783 MSATs genotyped on this sample panel. Of the 783 MSATs, 78 of these markers displayed values ≥0.1, and were selected for further analyses. Pairwise F_st_ values of these 78 markers (**Table 1A**) indicate capacity to distinguish continental population groups. Progressively smaller sets of markers (**Table 1B–D**) were used for MSAT selection during each subsequent reduction in marker number prior to application of STRUCTURE and PCA (**Figure 2** **,** **Figure 3**).

**Table 1:** Pairwise F_st_ values for markers of the HGDP.

A		78 MSATs					B		48 MSATs				
	AF^a^	AM	CA	EA	EU	ME		AF	AM	CA	EA	EU	ME
**AF**							**AF**						
**AM**	0.398						**AM**	0.363					
**CA**	0.329	0.283					**CA**	0.283	0.264				
**EA**	0.316	0.197	0.179				**EA**	0.305	0.151	0.108			
**EU**	0.370	0.306	0.073	0.175			**EU**	0.221	0.255	0.066	0.155		
**ME**	0.387	0.283	0.048	0.162	0.033		**ME**	0.251	0.244	0.052	0.121	0.029	
**OC**	0.391	0.299	0.291	0.233	0.367	0.373	**OC**	0.389	0.253	0.266	0.219	0.340	0.286

C		36MSATs					D		24MSATs				
	AF	AM	CA	EA	EU	ME		AF	AM	CA	EA	EU	ME
**AF**							**AF**						
**AM**	0.303						**AM**	0.211					
**CA**	0.161	0.222					**CA**	0.110	0.201				
**EA**	0.241	0.137	0.091				**EA**	0.202	0.092	0.083			
**EU**	0.189	0.197	0.052	0.133			**EU**	0.173	0.121	0.021	0.129		
**ME**	0.163	0.231	0.036	0.127	0.027		**ME**	0.121	0.173	0.017	0.099	0.010	
**OC**	0.321	0.273	0.201	0.133	0.173	0.232	**OC**	0.183	0.143	0.112	0.087	0.143	0.133

a Populations abbreviations are: AF, Africa; AM, Americas, CA, South Central Asia; EA, East Asia; EU, Europe; ME, Middle East; OC, Oceania.

Both PCA and STRUCTURE demonstrated that the 78 markers identified via F_st_ values differentiate the five continental population groups (**Figure 2** **,** **Figure 3A**). STRUCTURE analysis of the 78 MSAT set was performed with K = 2 through K = 12. Quantitative analysis of the results, using the StructureSum algorithm (see Materials and Methods), demonstrated that five population clusters (K = 5) is the best fit for the data. To determine whether a smaller subset of markers is sufficient to differentiate these 5 populations, the set of 78 MSATs was reduced in a step-wise fashion to 48, 36 and finally to 24 markers. Pairwise F_st_ analyses of the 48 and 36 marker sets both display (**Table 1A–C**) a similar capacity to distinguish among continental population groups and a limited capacity to discern among the more closely related population groups (e.g. European, Middle Eastern, and Central Asian). When the number of markers is reduced to 24 (**Table 1D**), the marker set displays large F_st_ values for comparisons of only the most divergent population groups (e.g. African vs. Oceanic).

The 3 smaller groupings of markers (48, 36 and 24 MSATs) were tested further using STRUCTURE and PCA. Analyses of the STRUCTURE data for the 48 and 36 MSAT panel indicated that five population groups (K = 5) are the best fit for both datasets, which effectively resolve the five major continental population clusters (**Figure 2**): Africa, Americas, Caucasian (Europe, Middle East and Central Asia), East Asia, and Oceania. In contrast, when the number of markers is reduced to 24 MSATs, only 4 (K = 4) population groups can be resolved. Africa, Americas and Caucasian (Europe, Middle East and Central Asia) can still be distinguished, but East Asia and Oceania cannot (**Figure 2**).

Similarly, PCA analyses (**Figure 3**) yield five distinct population clusters for all groupings except the set of 24 MSATs. In all cases the first and second PCs (PC1 and PC2) explain the majority of total variance in each case (e.g. 49% and 27% respectively for the 36 MSATs set). The addition of a third PC does not distinguish these five groups more effectively, nor does it allow differentiation of additional population clusters (**Figure S2**). Taken together, the results provide evidence that a minimal panel of 36 MSATs can be used to distinguish human ancestries from five major continental regions with the same efficiency as a larger panel of 78 MSATs. The panel of 36 MSATs that comprise this set of markers, listed in **Table S3**, has been termed CoAIMs, for Continental Ancestry Informative Markers.

### Experimental Validation of CoAIMs Using Established Population Samples

**Figure 4:**
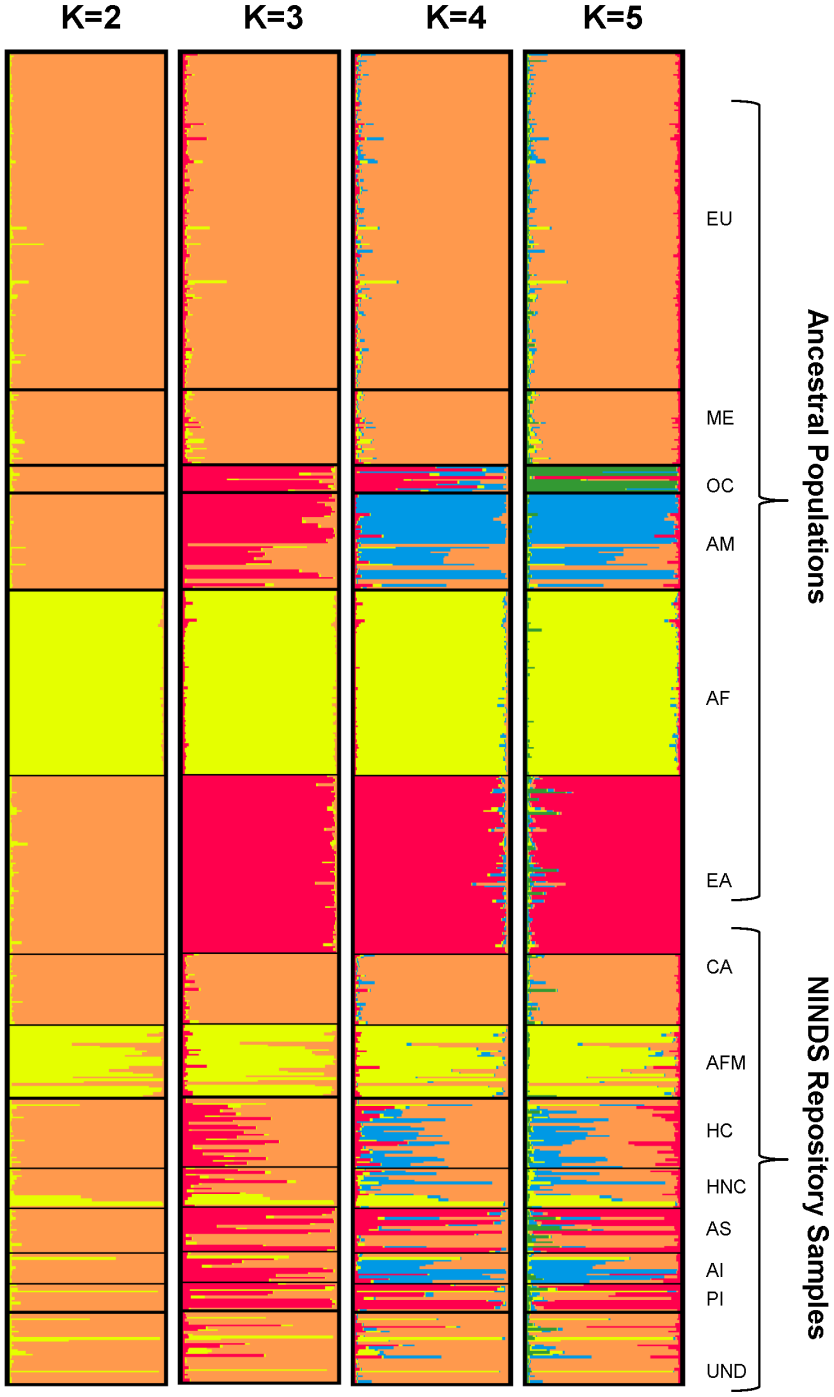
STRUCTURE Analysis of CoAIMs. The number of clusters assumed (K) is shown for each panel. Color assignments correspond to the continental group (cluster) with the largest membership in that cluster. Ancestral population groups from NHGRI and NIGMS (Ancestral Populations) include: Europe (EU), Middle East (ME), Oceania (OC), Americas (AM), Africa (AF) and East Asia (EA). Self-declared NINDS Repository groups (NINDS Repository Samples) include: Caucasian (CA), African American (AFM), Hispanic Caucasian (HC), Hispanic non-Caucasian (HNC), Asian (AS), American Indian (AI), Pacific Islander (PI), Mixed Race or Undeclared (UND).

The CoAIMs set of MSATs was identified using *in silico* analyses of datasets representing previously genotyped samples of the HGDP. To confirm further that CoAIMs can distinguish among the continental population groups, the 36 MSATs were optimized to be genotyped in three multiplex PCR reactions consisting of 12 markers each (see Materials and Methods).Using this scheme, we genotyped 710 NIGMS and NHGRI population control samples, determined pairwise F_st_ values (**Table 2**), and analyzed the data by STRUCTURE and PCA (**Figure 4** **–** **5**). This approach allowed us to independently test the effectiveness of CoAIMs for distinguishing among population groups in this independent set of population samples. It should be noted that populations from the South Central Asia, included in the HGDP, were not available from NIGMS and NHGRI, and are therefore not included here.

**Figure 5:**
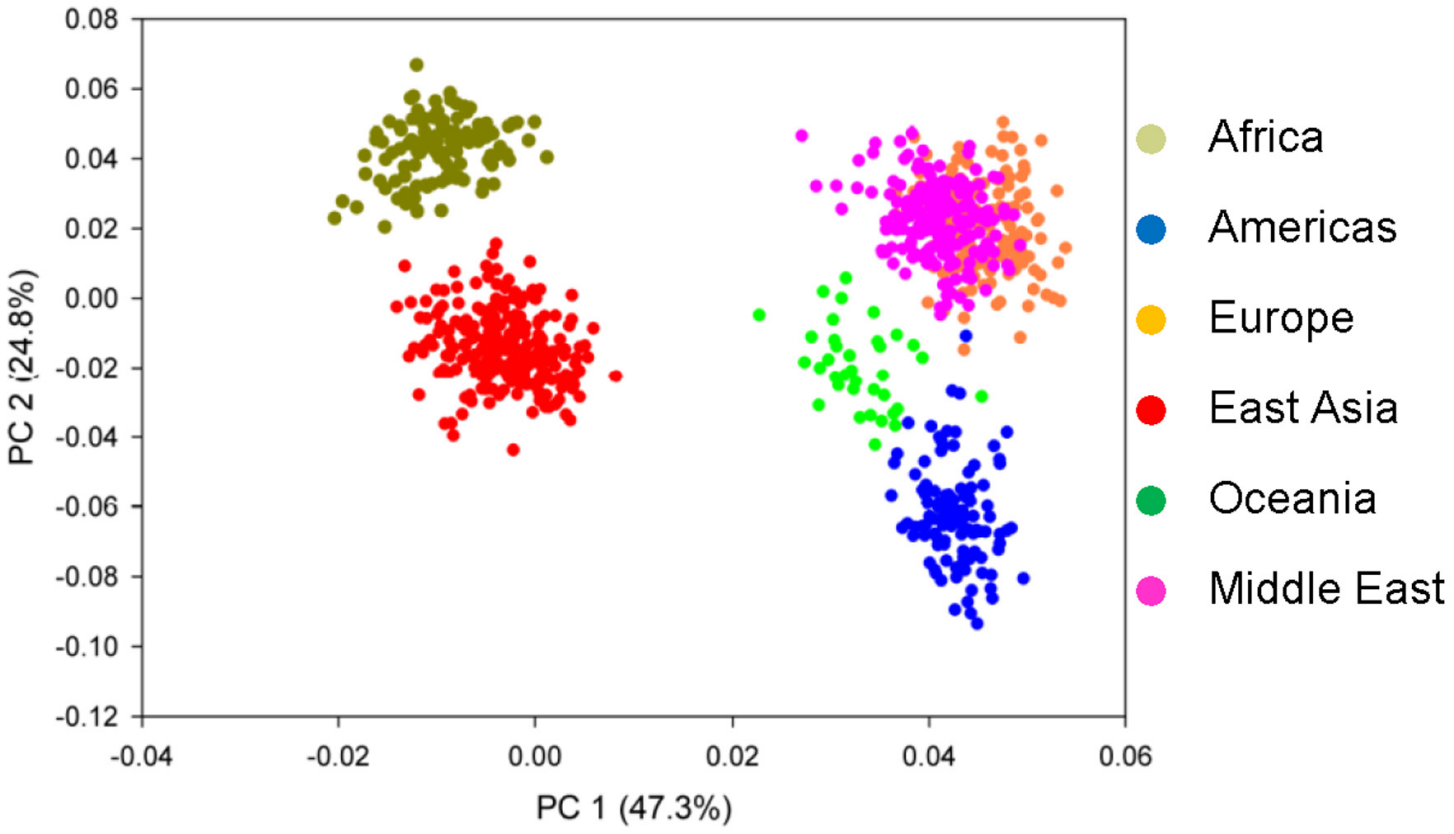
PCA Analysis of CoAIMs. The analysis used the same data set (Ancestral Populations) indicated in **Figure 3**. The population groups are shown by the color-coded symbols. The results for PC1 and PC2 are shown.

**Table 2:** Paired F_st_ Values for the 36 MSATs of the CoAIMs panel.

	AF	AM	EA	EU	ME	OC
**AF**						
**AM**	0.323					
**EA**	0.225	0.091				
**EU**	0.201	0.211	0.143			
**ME**	0.151	0.223	0.113	0.021		
**OC**	0.349	0.278	0.199	0.092	0.222	

**AF**, Africa; **AM**, Americas; **EA**, East Asia; **EU**, Europe; **ME**, Middle East; **OC**, Oceania.

Analyses of the genotype data from the NIGMS and NHGRI population samples that were obtained using CoAIMs confirmed high individual F_st_ values ranging from 0.252 to 0.110 (0.165±0.035; **Table S3**). Moreover, pairwise F_st_ analyses further demonstrate high intercontinental F_st_ values. Similar to the results obtained with HGDP data, the two closely related Caucasian populations from Europe and the Middle East displayed small pairwise F_st_ values (**Table 2**), suggesting limited power to distinguish between these two groups.

STRUCTURE was also used to evaluate how effectively the CoAIMs panel distinguishes among population groups. Genotype data for the set of 36 markers were analyzed under different K values, with the number of population groups (clusters) assumed ranging from two to twelve (K = 2 to K = 12). As the number of population clusters assumed, K, is increased from K = 2 to K = 5, so increases the number of distinguishable population groups (**Figure 4**). At a K = 5, five continental population groups are discerned: African, East Asian, Oceania, Native American, and Caucasian (Europe and Middle Eastern). StructureSum (see Materials and Methods) revealed that a maximum of five population groups (K = 5) explains the data, as was the case for STRUTCTURE and StructureSum analyses of the HGDP dataset (**Figure 3**). Stepwise increases in the number of assumed population clusters (K = 6 to K = 12) does not resolve additional population clusters (data not shown).

PCA analyses (**Figure 5**) confirmed that five distinct continental population clusters are identified by CoAIMs. The top 2 PCs explain the large majority of the variance (47.3% and 24.8% respectively, **Figure 5**), and the addition of the third PC (8.6% of the total variance) does not increase or decrease the number of population clusters that are distinguished (**Figure S3A–B**). Taken together, results obtained using 710 NIGMS and NHGRI ancestral population samples allowed independent verification of the results obtained via *in silico* experiments using a publically available HGDP genotype dataset.

### CoAIMs Can Assess Continental Admixture Proportions in Samples of Self-Declared Ancestry

DNA samples from the NINDS Human Genetics DNA and Cell Line Repository were examined with CoAIMs to determine continental ancestral proportions. Currently, more than 29,000 samples from diverse ethnic groups have been banked and more than 4,000 have been used in GWAS of Parkinson's disease, ALS, and other disorders [43]–[49]. Approximately 20% of the NINDS Repository samples are of non-Caucasian self-identified ancestry and therefore represent a valuable resource for studies of heritable disease in under-represented minority populations. We hypothesized that CoAIMs could provide parsimonious evaluation of genetic ancestry that, for example, would allow better matching between cases and controls. Thus, we applied CoAIMs to samples from the NINDS Repository with the following self-declared ancestries: Caucasian (n = 92), African American (n = 92), Asian (n = 20), Caucasian-Hispanic (n = 92), non-Caucasian Hispanic (n = 20), Pacific Islander (n = 14), American Indian (n = 25) and undeclared or mixed race (n = 30). Results were analyzed using STRUCTURE to assign continental admixture proportions. The NIGMS and NHGRI ancestral populations (**Figure 4**) were used as references to determine continental ancestral proportions for the samples of the NINDS Repository.

**Figure 6:**
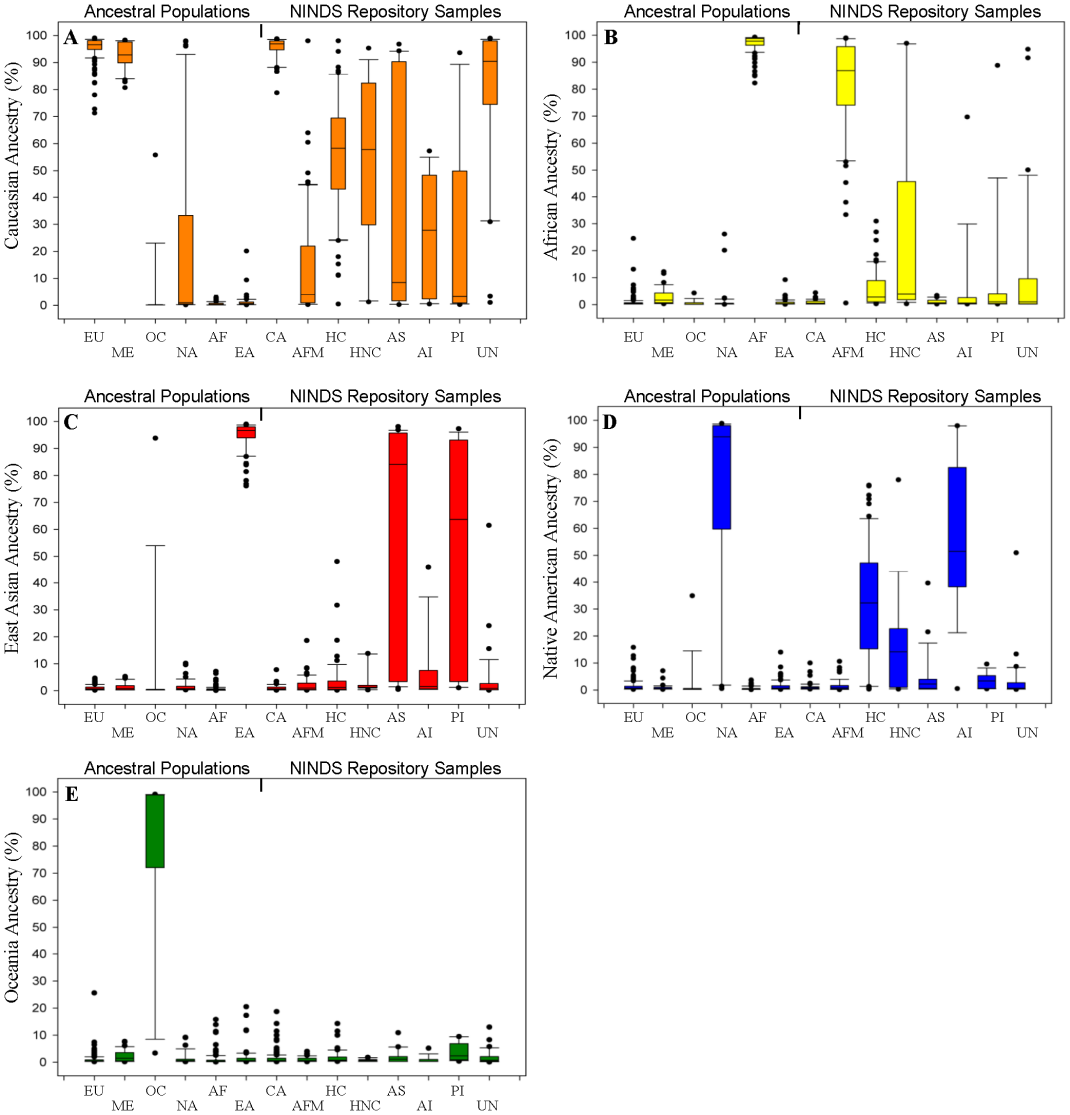
Ancestry Proportions of Admixed and Non-Admixed Populations of the NINDS Repository Using the CoAIMs. Box plot of ancestry proportions derived from STRUCTURE (K = 5). The lengths of the boxes are the inter-quartile ranges (25^th^–75^th^) with the median value indicated by the bar. The whiskers represent the value within 10^th^ and 90^th^ percentiles quartile of the lower and upper ranges and dots are extreme outlying samples. Panels A–E quantify percent (along the y axis) of the five continental ancestries measured using CoAIMs versus reported group indicated (along the x-axis; Ancestral Populations, NINDS Repository Samples) as follows: (**A**) Caucasian ancestry, (**B**) African ancestry, (**C**) East Asian ancestry, (**D**) and Native American ancestry, (**E**) Oceania ancestry. Ancestral Populations and NINDS Repository Samples are arranged across the x-axes as follows: Europe (EU), Middle East (ME), Oceania (OC), Americas (NA), Africa (AF), East Asia (EA), Caucasian (CA), African American (AFM), Caucasian Hispanic (HC), non-Caucasian Hispanic (HNC), Asian (11), American Indian (AI), Pacific Islander (PI), and Undeclared (UN; includes samples indicated as being of more than one race).

Based on previous studies of self-declared ancestry [5], [13], we expected that of the eight NINDS Repository groups analyzed, Caucasians represent the only self-declared group that displays relatively little, if any, continental admixture after analysis of CoAIMs (**Figure 4**, NINDS Repository samples). Admixture proportion estimates support this observation (**Figure 6**). Interestingly, all self-declared non-European groups in the NINDS Repository display measurable Caucasian ancestry (**Figure 6A**) and, in some instances, contributions from other ancestral groups (**Figure 6B–E**). For example, CoAIMs verified that self-declared African Americans are, on average, mostly of sub-Saharan African ancestry (0.81%±0.20%, n = 92, **Figure 6B**), while also displaying Caucasian admixture (0.14%±0.20%, **Figure 6A**). We did not detect ancestral contribution from any of the other continental groups in the African American sample set. Self-declared American Indians (n = 25) displayed both Native American (0.53%±0.33%) and Caucasian ancestry (0.32%±0.29%); a result consistent with other studies [50], [51]. Similarly, self-declared Asian individuals displayed range of proportions between East Asian and Caucasian ancestries (**Figures 4** **and** **6**). Interestingly, self-declared Pacific Islander subjects, displayed larger proportions of East Asian ancestries than Oceanic ancestry (**Figures 4** **and** **6**). Finally, individuals that did not self-declare an ancestry (n = 30), or declared to be more than one race, frequently displayed a large proportion of Caucasian ancestry (0.77%±0.03%, **Figure 6A**).

Hispanic populations are known to have Caucasian, African, and Native American ancestries [50], [51]. Two separate Hispanic populations were examined here, those of self-declared Caucasian (n = 92) and non-Caucasian ethnicities (n = 20) from North America. The self-declared Hispanic non-Caucasian group displayed an African ancestry proportion of 0.24%±0.35% (also see **Figure 6B**), and Native American ancestry proportion of 0.18%±0.20% (also see **Figure 6D**). Conversely, the self-declared Hispanic Caucasian group displayed an African ancestry proportion of 0.04%±0.08 (**Figure 6B**), and a Native American ancestry proportions of 0.33%±0.21 (**Figure 6D**). T-tests demonstrated that difference in African proportions between the Hispanic Caucasian and the Hispanic non-Caucasian groups is highly significant (p<0.001), while the difference in the Native American proportions approaches statistical significance (p = 0.05). As expected, these two groups displayed indistinguishable proportions of Caucasian genetic ancestry (0.53%±0.33 and 0.55%±0.22, respectively, **Figure 6A**; confirmed by t-test [p = 0.99]). Taken together, these results demonstrated that the CoAIMs panel is suitable for determining continental ancestry and admixture proportions for non-admixed and admixed individuals.

## Discussion

The current study was performed to develop a set of AIMs for efficiently and reliably discerning among continental population groups. The results presented here demonstrate the development and utility of CoAIMs, a 36-MSAT panel that measures genetic ancestry. This MSAT-based approach uses a routine and cost-effective genotyping methodology. CoAIMs can be used to determine continental ancestry and admixture, as well as to cluster individuals from a cohort into discrete ancestry groups to control for the confounding effects of population stratification in genetic studies. The clustering patterns observed for the five major continental ancestries studied here are similar to those obtained in other studies using larger sets of markers [24], [27], [52], [53]. Furthermore, the ancestral proportions that we measured confirm the inadequacies of relying solely on self-declared ancestry, and suggest that caution should be used when studying these admixed population groups.

Both SNPs and MSATs have been used to determine genetic ancestry. SNP AIM panels that require large numbers of markers (>100) to determine continental ancestry have been described [16], [21], [22], [54]. The large number of SNPs needed reflects the bi-allelic nature of these markers in that a single SNP can distinguish between a maximum of two ancestry populations [55]. In contrast, MSAT AIMs are multi-allelic, with each marker distinguishing among multiple population groups and have a greater potential for higher information content than SNP [55]. The results presented here demonstrate the usefulness of a targeted MSAT panel for detecting genetic ancestry. The 36 MSAT markers that comprise CoAIMs were chosen based on their high F_st_ values, and STRUCTURE and PCA results of genotyping parental population samples confirm that these markers can distinguish five discrete population groups: Caucasian (European and Middle Eastern), African, East Asian, Native American and Pacific Islander. Interestingly, our primary decision in marker choice was based upon high informativeness value (F_st_), and not the specific repeat structure of the MSAT (i.e. dinucleotide vs. tetranucleotide). Previous studies have shown that dinucleotide repeats are more stable and are more suitable for determining population structure [55], [56]. The CoAIMs panel is comprised of 33 MSATs with dinucleotide repeats, 2 with tetranucleotide repeats, and 1 with trinucleotide repeats.

We hypothesized that CoAIMs can be used to measure continental admixture proportions in subjects of admixed descent. To test this hypothesis we examined individuals from heterogeneous population groups, utilizing DNA samples banked in the NINDS Repository. Analyses of African American individuals demonstrated admixture proportions (African ancestry 0.81±0.20 and 0.14±0.20 Caucasian ancestry) similar to those observed using SNP AIMs panels consisting of a large numbers of markers [15], [21], [51]. However one individual (ND09555) submitted to the repository with race reported as African American was identified using CoAIMs to have nearly 100% Caucasian ancestry. Recontact with the submitter of the biospecimen revealed that the initially reported race was in error. This finding illustrates the utility of the CoAIMs assay in large biobanking efforts.

In addition to African Americans, individuals from two separate Hispanic populations were examined representing those self-declared as Caucasian, and those self-declared as non-Caucasian. Genetic studies of Hispanic populations have displayed a trihybrid ancestral population structure between Caucasian, Native American and African populations. The proportions of these three ancestral population groups varied greatly [15]. Our analyses of a small number of samples from these two Hispanic population groups reflected this complex population structure. While in both groups, Caucasian and Native American ancestries were the predominant ancestral groups, the non-Caucasian Hispanic overall had less Native American and increased amounts of African ancestries. Significant differences in the proportions of African (p = 0.0001) and Native Americans (p = 0.542) ancestries were observed between the two groups. This difference in ancestral proportions between the two groups may reflect the location of sample collection. Hispanic individuals from the Eastern United states tend to have higher European and African ancestry than those from the Western United States [15]. Similarly, Hispanic individuals of Cuban and Puerto Rican descent tend to have predominant Caucasian and African ancestry and minor amounts of Native American ancestry [15]. Though the number of Hispanic individuals studied was small, these data suggest that CoAIMs has the ability to capture the complex genetic heterogeneity present within Hispanic population groups.

Measurements of genetic continental ancestral proportions in samples of self-declared ancestry by CoAIMs is made possible by the inclusion of ancestral population groups. In the analyses performed here, individuals from the NIGMS and NHGRI Repositories were used as parental population reference groups. As expected, NIGMS and NHGRI samples from the Caucasian, African and East Asian populations separated into highly discrete population clusters. This is in contrast to the Native American ancestral population group which displayed individuals with measurable proportions of European ancestry. These individuals were identified as Mexican and Mexican Indian descent from the NIGMS Human Population Collection. Previous genetic studies of these population groups have shown them to have European admixture [50], [51], [57], which may explain the Caucasian ancestry component observed using CoAIMs. Analyses of the data with these subjects removed did not affect the estimated admixture proportions the NINDS Repository self-reported ancestry samples (data not shown). Thus the examination of parental population groups by CoAIMs is critical to accurately assess continental ancestral proportions. While CoAIMs was specifically developed to discern among the major continental population groups, and our results indicate this, the further differentiation of intracontinental populations may be possible with larger marker sets.

The complex genetic heterogeneity that exists within admixed populations often confounds genetic association studies, a major application of AIMs is to control for these adverse effects of population stratification in GWAS. CoAIMs can be readily applied to GWAS as an efficient method to adjust for the differences in continental ancestry between cases and controls. This panel can be used to include or exclude subjects from a study cohort based upon continental ancestral proportions. This would be particularly effective for studies involving Hispanic or African American populations that contain wide ranges of admixture among ancestral populations. Additionally, applying CoAIMs prior to performing whole-genome genotyping can eliminate the expense of high throughput SNP genotyping of extraneous samples. Ultimately, the use of CoAIMs can help facilitate a better understanding of the significance of existing GWAS data as well as future genetic studies in both Caucasian and non-Caucasian populations.

Finally, it is becoming increasingly evident that many health-related traits are influenced by an individual's genetic ancestry. For example, increasing proportions of Native American ancestry have been associated with milder asthma among Mexican Americans [58]. In a recent study among Puerto Ricans, African ancestry was negatively associated with type-2 diabetes and cardiovascular disease and positively correlated with hypertension [59]. Similarly, a higher percentage of Caucasian ancestry in Hispanic populations has been significantly associated with increased breast cancer risk [8]. Therefore, the ability to make inferences about an individual's ancestral proportions could contribute to disease susceptibility estimates. Thus, the ancestral proportion derived from CoAIMs provides significant benefits in such efforts.

## Supporting Information

Figure S1 PCA Plots of the Top Three PCs of the set of 19 MSATs (ABI identifiler and Coriell 6-plex). Plot of PC1 vs. PC3 (A) which captures 56.1% and 7.2% of the total variation of the data. Plot of PC2 vs. PC3 (B) which captures 22.9% and 7.2% of the total variation of the data.
(0.19 MB TIF)

Figure S2 PCA Plots From Analysis of the HGDP MSATs. (A) PC1 vs. PC3 and (B) PC2 vs. PC3 for the 78 MSAT marker set. (C) PC1 vs. PC3 and (D) PC2 vs. PC3 for the 48 MSAT marker set. (E) PC1 vs. PC3 and (F) PC2 vs. PC3 for the36 MSAT marker set. (G) PC1 vs. PC3 and (H) PC2 vs. PC3 for the 24 MSAT marker set. In all cases, the addition of the third PC does not allow for further separation of population cluster.
(0.16 MB TIF)

Figure S3 PCA Plots of CoAIMs with the NIGMS and NHGRI Population Samples. Plot of PC1 vs. PC3 (A) and PC2 vs. PC3 (B) of the CoAIMs panel with the NIGMS and NHGRI population samples.
(0.17 MB TIF)

Table S1 Samples from the NIGMS, NHGRI and NINDS Cell Repositories used.
(0.08 MB DOCX)

Table S2 The 19 MSATs of the ABI Identifiler and Coriell Identity Mapping kit and their Fst values.
(0.04 MB DOC)

Table S3 MSATs of the CoAIM panel.
(0.02 MB DOCX)

Table S4 PCR conditions of the 3 multiplex PCRs for CoAIMs.
(0.01 MB DOCX)
