# Supplementary figures and images for: CoAIMs: A Cost-Effective Panel of Ancestry Informative Markers for Determining Continental Origins
Source: PLoS One. 2010 Oct 15;5(10):e13443. doi: 10.1371/journal.pone.0013443 (PMC2955551; doi:10.1371/journal.pone.0013443)

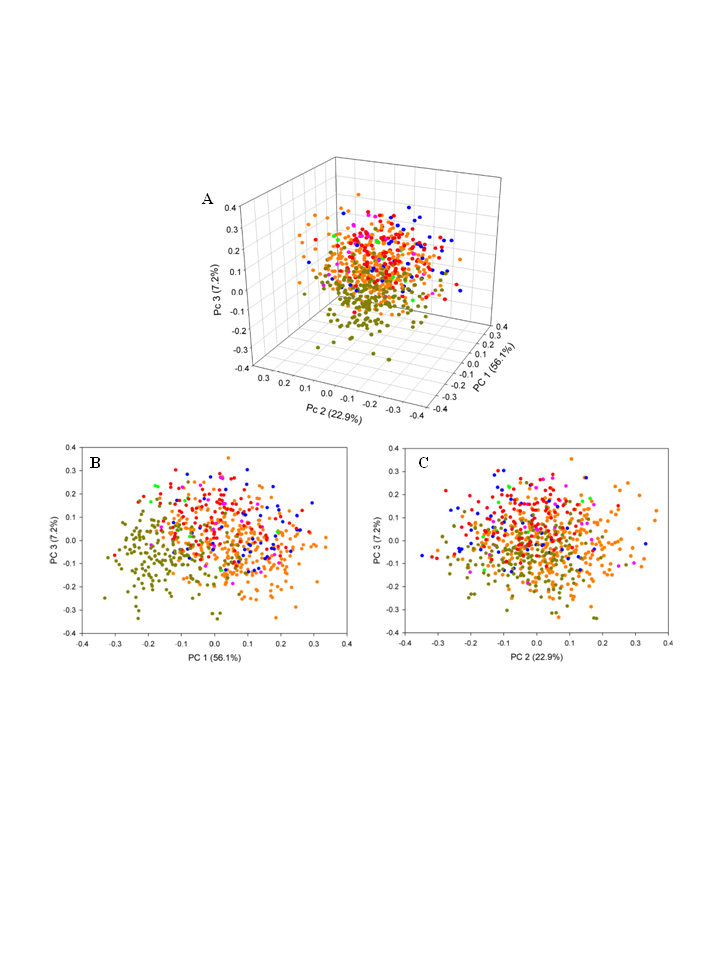

Supplement: Figure S1 — PCA Plots of the Top Three PCs of the set of 19 MSATs (ABI identifiler and Coriell 6-plex). Plot of PC1 vs. PC3 (A) which captures 56.1% and 7.2% of the total variation of the data. Plot of PC2 vs. PC3 (B) which captures 22.9% and 7.2% of the total variation of the data. (0.19 MB TIF) [file pone.0013443.s001.tif]

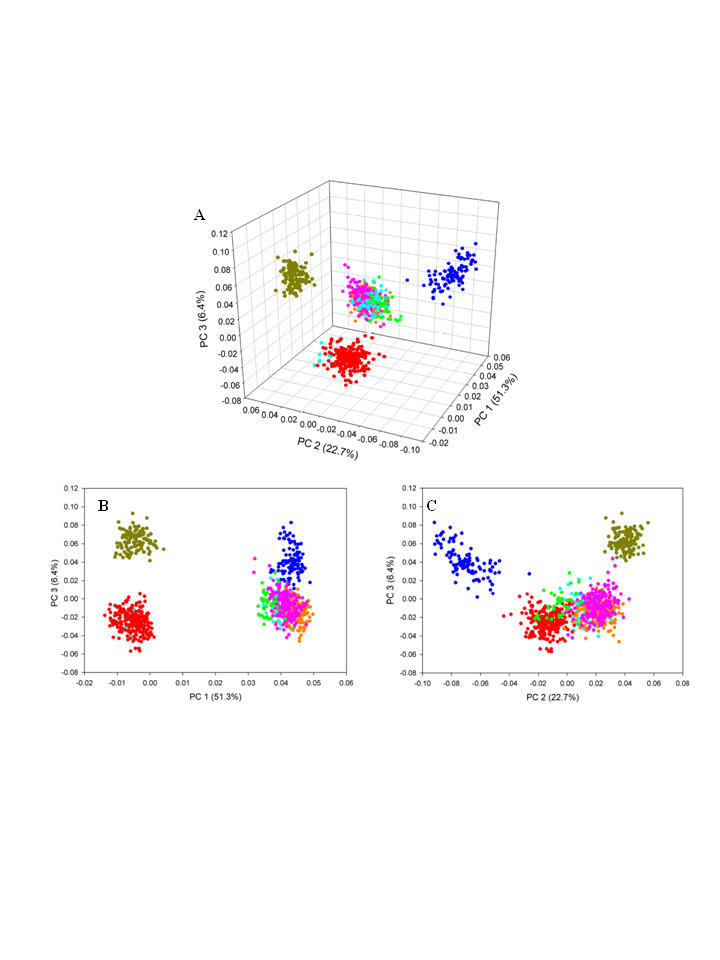

Supplement: Figure S2 — PCA Plots From Analysis of the HGDP MSATs. (A) PC1 vs. PC3 and (B) PC2 vs. PC3 for the 78 MSAT marker set. (C) PC1 vs. PC3 and (D) PC2 vs. PC3 for the 48 MSAT marker set. (E) PC1 vs. PC3 and (F) PC2 vs. PC3 for the36 MSAT marker set. (G) PC1 vs. PC3 and (H) PC2 vs. PC3 for the 24 MSAT marker set. In all cases, the addition of the third PC does not allow for further separation of population cluster. (0.16 MB TIF) [file pone.0013443.s002.tif]

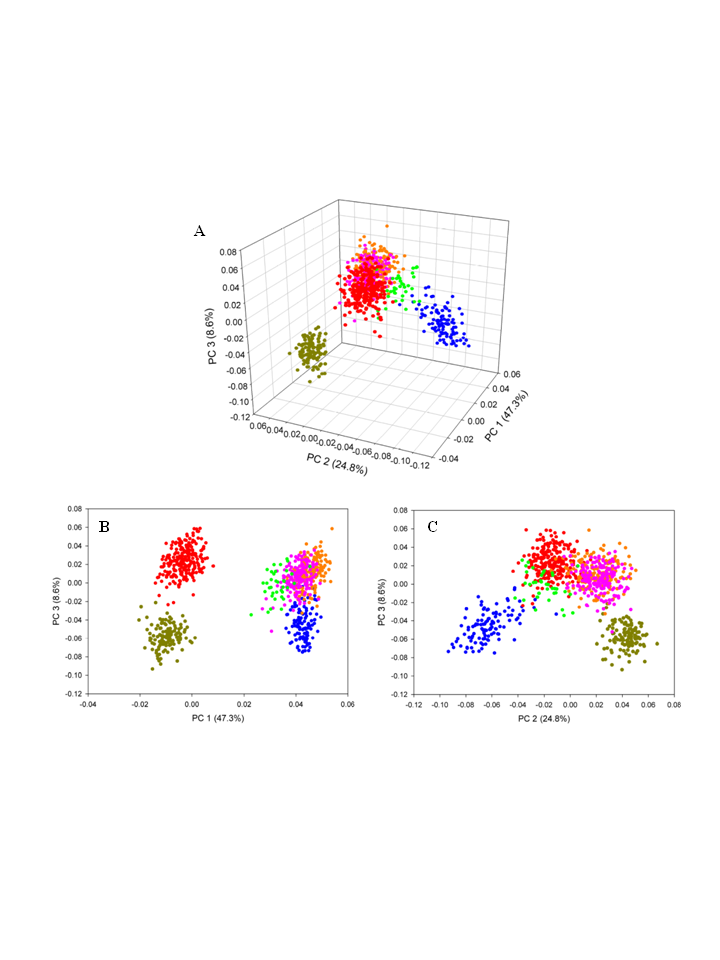

Supplement: Figure S3 — PCA Plots of CoAIMs with the NIGMS and NHGRI Population Samples. Plot of PC1 vs. PC3 (A) and PC2 vs. PC3 (B) of the CoAIMs panel with the NIGMS and NHGRI population samples. (0.17 MB TIF) [file pone.0013443.s003.tif]
