# Supplementary material for: CoAIMs: A Cost-Effective Panel of Ancestry Informative Markers for Determining Continental Origins
Source: PLoS One. 2010 Oct 15;5(10):e13443. doi: 10.1371/journal.pone.0013443 (PMC2955551; doi:10.1371/journal.pone.0013443)
Supplement: Table S1 — Samples from the NIGMS, NHGRI and NINDS Cell Repositories used. (0.08 MB DOCX) [file pone.0013443.s004.docx]

| **Coriell Sample ID** | **Continental population** | **population** | **Coriell Cell Repository^*^** |
| --- | --- | --- | --- |
| NA06985 | Europe | CEPH | NIGMS |
| NA06986 | Europe | CEPH | NIGMS |
| NA06993 | Europe | CEPH | NIGMS |
| NA06994 | Europe | CEPH | NIGMS |
| NA07000 | Europe | CEPH | NIGMS |
| NA07022 | Europe | CEPH | NIGMS |
| NA07031 | Europe | CEPH | NIGMS |
| NA07034 | Europe | CEPH | NIGMS |
| NA07037 | Europe | CEPH | NIGMS |
| NA07045 | Europe | CEPH | NIGMS |
| NA07051 | Europe | CEPH | NIGMS |
| NA07055 | Europe | CEPH | NIGMS |
| NA07056 | Europe | CEPH | NIGMS |
| NA07345 | Europe | CEPH | NIGMS |
| NA07346 | Europe | CEPH | NIGMS |
| NA07347 | Europe | CEPH | NIGMS |
| NA07357 | Europe | CEPH | NIGMS |
| NA07435 | Europe | CEPH | NIGMS |
| NA10859 | Europe | CEPH | NIGMS |
| NA11829 | Europe | CEPH | NIGMS |
| NA11830 | Europe | CEPH | NIGMS |
| NA11831 | Europe | CEPH | NIGMS |
| NA11832 | Europe | CEPH | NIGMS |
| NA11839 | Europe | CEPH | NIGMS |
| NA11840 | Europe | CEPH | NIGMS |
| NA11843 | Europe | CEPH | NIGMS |
| NA11881 | Europe | CEPH | NIGMS |
| NA11882 | Europe | CEPH | NIGMS |
| NA11891 | Europe | CEPH | NIGMS |
| NA11892 | Europe | CEPH | NIGMS |
| NA11893 | Europe | CEPH | NIGMS |
| NA11894 | Europe | CEPH | NIGMS |
| NA11917 | Europe | CEPH | NIGMS |
| NA11918 | Europe | CEPH | NIGMS |
| NA11919 | Europe | CEPH | NIGMS |
| NA11920 | Europe | CEPH | NIGMS |
| NA11930 | Europe | CEPH | NIGMS |
| NA11931 | Europe | CEPH | NIGMS |
| NA11992 | Europe | CEPH | NIGMS |
| NA11993 | Europe | CEPH | NIGMS |
| NA11994 | Europe | CEPH | NIGMS |
| NA11995 | Europe | CEPH | NIGMS |
| NA12003 | Europe | CEPH | NIGMS |
| NA12004 | Europe | CEPH | NIGMS |
| NA12005 | Europe | CEPH | NIGMS |
| NA12006 | Europe | CEPH | NIGMS |
| NA12043 | Europe | CEPH | NIGMS |
| NA12044 | Europe | CEPH | NIGMS |
| NA12045 | Europe | CEPH | NIGMS |
| NA12056 | Europe | CEPH | NIGMS |
| NA12057 | Europe | CEPH | NIGMS |
| NA12058 | Europe | CEPH | NIGMS |
| NA12144 | Europe | CEPH | NIGMS |
| NA12145 | Europe | CEPH | NIGMS |
| NA12146 | Europe | CEPH | NIGMS |
| NA12154 | Europe | CEPH | NIGMS |
| NA12155 | Europe | CEPH | NIGMS |
| NA12156 | Europe | CEPH | NIGMS |
| NA12234 | Europe | CEPH | NIGMS |
| NA12236 | Europe | CEPH | NIGMS |
| NA12239 | Europe | CEPH | NIGMS |
| NA12248 | Europe | CEPH | NIGMS |
| NA12249 | Europe | CEPH | NIGMS |
| NA12264 | Europe | CEPH | NIGMS |
| NA12272 | Europe | CEPH | NIGMS |
| NA12273 | Europe | CEPH | NIGMS |
| NA12274 | Europe | CEPH | NIGMS |
| NA12275 | Europe | CEPH | NIGMS |
| NA12282 | Europe | CEPH | NIGMS |
| NA12283 | Europe | CEPH | NIGMS |
| NA12286 | Europe | CEPH | NIGMS |
| NA12287 | Europe | CEPH | NIGMS |
| NA12340 | Europe | CEPH | NIGMS |
| NA12341 | Europe | CEPH | NIGMS |
| NA12342 | Europe | CEPH | NIGMS |
| NA12343 | Europe | CEPH | NIGMS |
| NA12347 | Europe | CEPH | NIGMS |
| NA12348 | Europe | CEPH | NIGMS |
| NA12383 | Europe | CEPH | NIGMS |
| NA12399 | Europe | CEPH | NIGMS |
| NA12400 | Europe | CEPH | NIGMS |
| NA12413 | Europe | CEPH | NIGMS |
| NA12414 | Europe | CEPH | NIGMS |
| NA12489 | Europe | CEPH | NIGMS |
| NA12546 | Europe | CEPH | NIGMS |
| NA12716 | Europe | CEPH | NIGMS |
| NA12717 | Europe | CEPH | NIGMS |
| NA12718 | Europe | CEPH | NIGMS |
| NA12748 | Europe | CEPH | NIGMS |
| NA12749 | Europe | CEPH | NIGMS |
| NA12750 | Europe | CEPH | NIGMS |
| NA12751 | Europe | CEPH | NIGMS |
| NA12760 | Europe | CEPH | NIGMS |
| NA12761 | Europe | CEPH | NIGMS |
| NA12762 | Europe | CEPH | NIGMS |
| NA12763 | Europe | CEPH | NIGMS |
| NA12775 | Europe | CEPH | NIGMS |
| NA12776 | Europe | CEPH | NIGMS |
| NA12777 | Europe | CEPH | NIGMS |
| NA12778 | Europe | CEPH | NIGMS |
| NA12812 | Europe | CEPH | NIGMS |
| NA12813 | Europe | CEPH | NIGMS |
| NA12814 | Europe | CEPH | NIGMS |
| NA12815 | Europe | CEPH | NIGMS |
| NA12827 | Europe | CEPH | NIGMS |
| NA12828 | Europe | CEPH | NIGMS |
| NA12829 | Europe | CEPH | NIGMS |
| NA12830 | Europe | CEPH | NIGMS |
| NA12842 | Europe | CEPH | NIGMS |
| NA12843 | Europe | CEPH | NIGMS |
| NA12872 | Europe | CEPH | NIGMS |
| NA12873 | Europe | CEPH | NIGMS |
| NA12874 | Europe | CEPH | NIGMS |
| NA12875 | Europe | CEPH | NIGMS |
| NA12889 | Europe | CEPH | NIGMS |
| NA12890 | Europe | CEPH | NIGMS |
| NA12892 | Europe | CEPH | NIGMS |
| NA13617 | Europe | Krasnodar | NIGMS |
| NA13618 | Europe | Krasnodar | NIGMS |
| NA13619 | Europe | Krasnodar | NIGMS |
| NA13620 | Europe | Krasnodar | NIGMS |
| NA13622 | Europe | Krasnodar | NIGMS |
| NA13623 | Europe | Krasnodar | NIGMS |
| NA13624 | Europe | Krasnodar | NIGMS |
| NA13625 | Europe | Krasnodar | NIGMS |
| NA13626 | Europe | Krasnodar | NIGMS |
| NA13820 | Europe | Zversky | NIGMS |
| NA13838 | Europe | Zversky | NIGMS |
| NA13849 | Europe | Zversky | NIGMS |
| NA13852 | Europe | Zversky | NIGMS |
| NA13876 | Europe | Zversky | NIGMS |
| NA13877 | Europe | Zversky | NIGMS |
| NA13911 | Europe | Zversky | NIGMS |
| NA13912 | Europe | Zversky | NIGMS |
| NA13913 | Europe | Zversky | NIGMS |
| NA13914 | Europe | Zversky | NIGMS |
| NA15199 | Europe | Hungarian | NIGMS |
| NA15200 | Europe | Hungarian | NIGMS |
| NA15201 | Europe | Hungarian | NIGMS |
| NA15202 | Europe | Hungarian | NIGMS |
| NA15204 | Europe | Hungarian | NIGMS |
| NA15205 | Europe | Hungarian | NIGMS |
| NA15206 | Europe | Hungarian | NIGMS |
| NA15207 | Europe | Hungarian | NIGMS |
| NA15208 | Europe | Hungarian | NIGMS |
| NA15724 | Europe | Czechoslovakian | NIGMS |
| NA15725 | Europe | Czechoslovakian | NIGMS |
| NA15726 | Europe | Czechoslovakian | NIGMS |
| NA15727 | Europe | Czechoslovakian | NIGMS |
| NA15728 | Europe | Czechoslovakian | NIGMS |
| NA15729 | Europe | Czechoslovakian | NIGMS |
| NA15730 | Europe | Czechoslovakian | NIGMS |
| NA15731 | Europe | Czechoslovakian | NIGMS |
| NA15732 | Europe | Czechoslovakian | NIGMS |
| NA15733 | Europe | Czechoslovakian | NIGMS |
| NA15755 | Europe | Icelandic | NIGMS |
| NA15756 | Europe | Icelandic | NIGMS |
| NA15757 | Europe | Icelandic | NIGMS |
| NA15758 | Europe | Icelandic | NIGMS |
| NA15759 | Europe | Icelandic | NIGMS |
| NA15760 | Europe | Icelandic | NIGMS |
| NA15761 | Europe | Icelandic | NIGMS |
| NA15762 | Europe | Icelandic | NIGMS |
| NA15763 | Europe | Icelandic | NIGMS |
| NA15764 | Europe | Icelandic | NIGMS |
| NA15765 | Europe | Icelandic | NIGMS |
| NA15766 | Europe | Icelandic | NIGMS |
| NA15883 | Europe | Basque | NIGMS |
| NA15884 | Europe | Basque | NIGMS |
| NA15885 | Europe | Basque | NIGMS |
| NA15886 | Europe | Basque | NIGMS |
| NA15887 | Europe | Basque | NIGMS |
| NA16185 | Europe | Basque | NIGMS |
| NA16186 | Europe | Basque | NIGMS |
| NA16188 | Europe | Basque | NIGMS |
| NA16189 | Europe | Basque | NIGMS |
| NA16190 | Europe | Basque | NIGMS |
| NA17001 | Europe | Northern European | NIGMS |
| NA17002 | Europe | Northern European | NIGMS |
| NA17003 | Europe | Northern European | NIGMS |
| NA17004 | Europe | Northern European | NIGMS |
| NA17005 | Europe | Northern European | NIGMS |
| NA17006 | Europe | Northern European | NIGMS |
| NA17007 | Europe | Northern European | NIGMS |
| NA17008 | Europe | Northern European | NIGMS |
| NA17009 | Europe | Northern European | NIGMS |
| NA17010 | Europe | Northern European | NIGMS |
| NA17091 | Europe | Iberians | NIGMS |
| NA17092 | Europe | Iberians | NIGMS |
| NA17093 | Europe | Iberians | NIGMS |
| NA17094 | Europe | Iberians | NIGMS |
| NA17095 | Europe | Iberians | NIGMS |
| NA17096 | Europe | Iberians | NIGMS |
| NA17097 | Europe | Iberians | NIGMS |
| NA17098 | Europe | Iberians | NIGMS |
| NA17099 | Europe | Iberians | NIGMS |
| NA17100 | Europe | Iberians | NIGMS |
| NA17323 | Europe | Italian | NIGMS |
| NA17370 | Europe | Greek | NIGMS |
| NA17371 | Europe | Greek | NIGMS |
| NA17372 | Europe | Greek | NIGMS |
| NA17373 | Europe | Greek | NIGMS |
| NA17374 | Europe | Greek | NIGMS |
| NA17375 | Europe | Greek | NIGMS |
| NA17376 | Europe | Greek | NIGMS |
| NA17377 | Europe | Greek | NIGMS |
| NA20502 | Europe | Toscani | NHGRI |
| NA20504 | Europe | Toscani | NHGRI |
| NA20505 | Europe | Toscani | NHGRI |
| NA20506 | Europe | Toscani | NHGRI |
| NA20508 | Europe | Toscani | NHGRI |
| NA20509 | Europe | Toscani | NHGRI |
| NA20510 | Europe | Toscani | NHGRI |
| NA20512 | Europe | Toscani | NHGRI |
| NA20515 | Europe | Toscani | NHGRI |
| NA20516 | Europe | Toscani | NHGRI |
| NA20517 | Europe | Toscani | NHGRI |
| NA20518 | Europe | Toscani | NHGRI |
| NA20519 | Europe | Toscani | NHGRI |
| NA20520 | Europe | Toscani | NHGRI |
| NA20521 | Europe | Toscani | NHGRI |
| NA20522 | Europe | Toscani | NHGRI |
| NA20524 | Europe | Toscani | NHGRI |
| NA20525 | Europe | Toscani | NHGRI |
| NA20527 | Europe | Toscani | NHGRI |
| NA20528 | Europe | Toscani | NHGRI |
| NA20529 | Europe | Toscani | NHGRI |
| NA20530 | Europe | Toscani | NHGRI |
| NA20531 | Europe | Toscani | NHGRI |
| NA20534 | Europe | Toscani | NHGRI |
| NA20535 | Europe | Toscani | NHGRI |
| NA20538 | Europe | Toscani | NHGRI |
| NA20539 | Europe | Toscani | NHGRI |
| NA20540 | Europe | Toscani | NHGRI |
| NA20541 | Europe | Toscani | NHGRI |
| NA20542 | Europe | Toscani | NHGRI |
| NA20543 | Europe | Toscani | NHGRI |
| NA20544 | Europe | Toscani | NHGRI |
| NA20581 | Europe | Toscani | NHGRI |
| NA20582 | Europe | Toscani | NHGRI |
| NA20585 | Europe | Toscani | NHGRI |
| NA20586 | Europe | Toscani | NHGRI |
| NA20588 | Europe | Toscani | NHGRI |
| NA20589 | Europe | Toscani | NHGRI |
| NA20752 | Europe | Toscani | NHGRI |
| NA20753 | Europe | Toscani | NHGRI |
| NA20754 | Europe | Toscani | NHGRI |
| NA20755 | Europe | Toscani | NHGRI |
| NA20756 | Europe | Toscani | NHGRI |
| NA20757 | Europe | Toscani | NHGRI |
| NA20758 | Europe | Toscani | NHGRI |
| NA20759 | Europe | Toscani | NHGRI |
| NA20760 | Europe | Toscani | NHGRI |
| NA20761 | Europe | Toscani | NHGRI |
| NA20765 | Europe | Toscani | NHGRI |
| NA20766 | Europe | Toscani | NHGRI |
| NA20768 | Europe | Toscani | NHGRI |
| NA20769 | Europe | Toscani | NHGRI |
| NA20770 | Europe | Toscani | NHGRI |
| NA20771 | Europe | Toscani | NHGRI |
| NA20772 | Europe | Toscani | NHGRI |
| NA20773 | Europe | Toscani | NHGRI |
| NA20774 | Europe | Toscani | NHGRI |
| NA20775 | Europe | Toscani | NHGRI |
| NA20778 | Europe | Toscani | NHGRI |
| NA20783 | Europe | Toscani | NHGRI |
| NA20785 | Europe | Toscani | NHGRI |
| NA20786 | Europe | Toscani | NHGRI |
| NA20787 | Europe | Toscani | NHGRI |
| NA20790 | Europe | Toscani | NHGRI |
| NA20792 | Europe | Toscani | NHGRI |
| NA20795 | Europe | Toscani | NHGRI |
| NA20796 | Europe | Toscani | NHGRI |
| NA20797 | Europe | Toscani | NHGRI |
| NA20798 | Europe | Toscani | NHGRI |
| NA20799 | Europe | Toscani | NHGRI |
| NA20800 | Europe | Toscani | NHGRI |
| NA20801 | Europe | Toscani | NHGRI |
| NA20802 | Europe | Toscani | NHGRI |
| NA20803 | Europe | Toscani | NHGRI |
| NA20804 | Europe | Toscani | NHGRI |
| NA20805 | Europe | Toscani | NHGRI |
| NA20806 | Europe | Toscani | NHGRI |
| NA20807 | Europe | Toscani | NHGRI |
| NA20808 | Europe | Toscani | NHGRI |
| NA20809 | Europe | Toscani | NHGRI |
| NA20810 | Europe | Toscani | NHGRI |
| NA20811 | Europe | Toscani | NHGRI |
| NA20812 | Europe | Toscani | NHGRI |
| NA20813 | Europe | Toscani | NHGRI |
| NA20815 | Europe | Toscani | NHGRI |
| NA20816 | Europe | Toscani | NHGRI |
| NA20818 | Europe | Toscani | NHGRI |
| NA20819 | Europe | Toscani | NHGRI |
| NA20826 | Europe | Toscani | NHGRI |
| NA20828 | Europe | Toscani | NHGRI |
| NA11521 | Middle East | Druze | NIGMS |
| NA11522 | Middle East | Druze | NIGMS |
| NA11523 | Middle East | Druze | NIGMS |
| NA11524 | Middle East | Druze | NIGMS |
| NA11525 | Middle East | Druze | NIGMS |
| NA22234 | Middle East | Ashkenazi Jewish | NIGMS |
| NA22235 | Middle East | Iranian Jewish | NIGMS |
| NA22299 | Middle East | Ashkenazi Jewish | NIGMS |
| NA22300 | Middle East | Ashkenazi Jewish | NIGMS |
| NA22301 | Middle East | Ashkenazi Jewish | NIGMS |
| NA22302 | Middle East | Ashkenazi Jewish | NIGMS |
| NA22303 | Middle East | Ashkenazi Jewish | NIGMS |
| NA22304 | Middle East | Ashkenazi Jewish | NIGMS |
| NA22305 | Middle East | Ashkenazi Jewish | NIGMS |
| NA22306 | Middle East | Ashkenazi Jewish | NIGMS |
| NA22307 | Middle East | Ashkenazi Jewish | NIGMS |
| NA22308 | Middle East | Ashkenazi Jewish | NIGMS |
| NA22309 | Middle East | Moroccan Jewish | NIGMS |
| NA22310 | Middle East | Moroccan Jewish | NIGMS |
| NA22311 | Middle East | Moroccan Jewish | NIGMS |
| NA22312 | Middle East | Moroccan Jewish | NIGMS |
| NA22313 | Middle East | Moroccan Jewish | NIGMS |
| NA22314 | Middle East | Moroccan Jewish | NIGMS |
| NA22315 | Middle East | Moroccan Jewish | NIGMS |
| NA22316 | Middle East | Moroccan Jewish | NIGMS |
| NA22317 | Middle East | Moroccan Jewish | NIGMS |
| NA22318 | Middle East | Moroccan Jewish | NIGMS |
| NA22319 | Middle East | Iranian Jewish | NIGMS |
| NA22320 | Middle East | Iranian Jewish | NIGMS |
| NA22321 | Middle East | Iranian Jewish | NIGMS |
| NA22322 | Middle East | Iranian Jewish | NIGMS |
| NA22324 | Middle East | Iranian Jewish | NIGMS |
| NA22325 | Middle East | Iranian Jewish | NIGMS |
| NA22326 | Middle East | Iranian Jewish | NIGMS |
| NA22327 | Middle East | Iranian Jewish | NIGMS |
| NA22328 | Middle East | Iranian Jewish | NIGMS |
| NA13597 | East Asia | Atayal (Taiwan) | NIGMS |
| NA13598 | East Asia | Atayal (Taiwan) | NIGMS |
| NA13599 | East Asia | Atayal (Taiwan) | NIGMS |
| NA13600 | East Asia | Atayal (Taiwan) | NIGMS |
| NA13601 | East Asia | Atayal (Taiwan) | NIGMS |
| NA13602 | East Asia | Atayal (Taiwan) | NIGMS |
| NA13603 | East Asia | Atayal (Taiwan) | NIGMS |
| NA13604 | East Asia | Atayal (Taiwan) | NIGMS |
| NA13605 | East Asia | Atayal (Taiwan) | NIGMS |
| NA13606 | East Asia | Atayal (Taiwan) | NIGMS |
| NA13607 | East Asia | Ami (Taiwan) | NIGMS |
| NA13608 | East Asia | Ami (Taiwan) | NIGMS |
| NA13609 | East Asia | Ami (Taiwan) | NIGMS |
| NA13610 | East Asia | Ami (Taiwan) | NIGMS |
| NA13611 | East Asia | Ami (Taiwan) | NIGMS |
| NA13612 | East Asia | Ami (Taiwan) | NIGMS |
| NA13613 | East Asia | Ami (Taiwan) | NIGMS |
| NA13614 | East Asia | Ami (Taiwan) | NIGMS |
| NA13615 | East Asia | Ami (Taiwan) | NIGMS |
| NA13616 | East Asia | Ami (Taiwan) | NIGMS |
| NA10539 | Oceania | Melanesian | NIGMS |
| NA10540 | Oceania | Melanesian | NIGMS |
| NA10541 | Oceania | Melanesian | NIGMS |
| NA10542 | Oceania | Melanesian | NIGMS |
| NA10543 | Oceania | Melanesian | NIGMS |
| NA17385 | Oceania | Pacific | NIGMS |
| NA17386 | Oceania | Pacific | NIGMS |
| NA17387 | Oceania | Pacific | NIGMS |
| NA17388 | Oceania | Pacific | NIGMS |
| NA17389 | Oceania | Pacific | NIGMS |
| NA17390 | Oceania | Pacific | NIGMS |
| NA17391 | Oceania | Pacific | NIGMS |
| NA10965 | Americas | Karitiana | NIGMS |
| NA10966 | Americas | Karitiana | NIGMS |
| NA10967 | Americas | Karitiana | NIGMS |
| NA10968 | Americas | Karitiana | NIGMS |
| NA10969 | Americas | Karitiana | NIGMS |
| NA10970 | Americas | Surui | NIGMS |
| NA10971 | Americas | Surui | NIGMS |
| NA10973 | Americas | Surui | NIGMS |
| NA10974 | Americas | Surui | NIGMS |
| NA10975 | Americas | Mayan | NIGMS |
| NA10976 | Americas | Mayan | NIGMS |
| NA10978 | Americas | Mayan | NIGMS |
| NA10979 | Americas | Mayan | NIGMS |
| NA11197 | Americas | Quechua | NIGMS |
| NA11198 | Americas | Quechua | NIGMS |
| NA11199 | Americas | Quechua | NIGMS |
| NA11200 | Americas | Quechua | NIGMS |
| NA11201 | Americas | Quechua | NIGMS |
| NA14308 | Americas | Pima | NIGMS |
| NA14309 | Americas | Pima | NIGMS |
| NA14310 | Americas | Pima | NIGMS |
| NA14311 | Americas | Pima | NIGMS |
| NA14313 | Americas | Pima | NIGMS |
| NA17061 | Americas | Mexican | NIGMS |
| NA17062 | Americas | Mexican | NIGMS |
| NA17063 | Americas | Mexican | NIGMS |
| NA17064 | Americas | Mexican | NIGMS |
| NA17065 | Americas | Mexican | NIGMS |
| NA17066 | Americas | Mexican | NIGMS |
| NA17067 | Americas | Mexican | NIGMS |
| NA17068 | Americas | Mexican | NIGMS |
| NA17069 | Americas | Mexican | NIGMS |
| NA17070 | Americas | Mexican | NIGMS |
| NA17301 | Americas | South America (Andes) | NIGMS |
| NA17302 | Americas | South America (Andes) | NIGMS |
| NA17303 | Americas | South America (Andes) | NIGMS |
| NA17304 | Americas | South America (Andes) | NIGMS |
| NA17305 | Americas | South America (Andes) | NIGMS |
| NA17306 | Americas | South America (Andes) | NIGMS |
| NA17307 | Americas | South America (Andes) | NIGMS |
| NA17308 | Americas | South America (Andes) | NIGMS |
| NA17309 | Americas | South America (Andes) | NIGMS |
| NA17310 | Americas | South America (Andes) | NIGMS |
| NA17311 | Americas | South America (Andes) | NIGMS |
| NA17312 | Americas | South America (Brazil) | NIGMS |
| NA17313 | Americas | South America (Brazil) | NIGMS |
| NA17314 | Americas | South America (Brazil) | NIGMS |
| NA17315 | Americas | South America (Brazil) | NIGMS |
| NA17316 | Americas | South America (Brazil) | NIGMS |
| NA17317 | Americas | South America (Brazil) | NIGMS |
| NA17318 | Americas | South America (Brazil) | NIGMS |
| NA17319 | Americas | South America (Brazil) | NIGMS |
| NA17320 | Americas | South America (Brazil) | NIGMS |
| NA10469 | Africa | Biaka Pygmy | NIGMS |
| NA10470 | Africa | Biaka Pygmy | NIGMS |
| NA10471 | Africa | Biaka Pygmy | NIGMS |
| NA10472 | Africa | Biaka Pygmy | NIGMS |
| NA10473 | Africa | Biaka Pygmy | NIGMS |
| NA10492 | Africa | Mbuti Pygmy | NIGMS |
| NA10493 | Africa | Mbuti Pygmy | NIGMS |
| NA10494 | Africa | Mbuti Pygmy | NIGMS |
| NA10495 | Africa | Mbuti Pygmy | NIGMS |
| NA10496 | Africa | Mbuti Pygmy | NIGMS |
| NA17341 | Africa | S. Sahara Africa | NIGMS |
| NA17342 | Africa | S. Sahara Africa | NIGMS |
| NA17343 | Africa | S. Sahara Africa | NIGMS |
| NA17344 | Africa | S. Sahara Africa | NIGMS |
| NA17345 | Africa | S. Sahara Africa | NIGMS |
| NA17346 | Africa | S. Sahara Africa | NIGMS |
| NA17347 | Africa | S. Sahara Africa | NIGMS |
| NA17348 | Africa | S. Sahara Africa | NIGMS |
| NA17349 | Africa | S. Sahara Africa | NIGMS |
| NA17378 | Africa | N. Sahara Africa | NIGMS |
| NA17379 | Africa | N. Sahara Africa | NIGMS |
| NA17380 | Africa | N. Sahara Africa | NIGMS |
| NA17381 | Africa | N. Sahara Africa | NIGMS |
| NA17382 | Africa | N. Sahara Africa | NIGMS |
| NA17383 | Africa | N. Sahara Africa | NIGMS |
| NA17384 | Africa | N. Sahara Africa | NIGMS |
| NA18486 | Africa | Yoruba | NHGRI |
| NA18487 | Africa | Yoruba | NHGRI |
| NA18489 | Africa | Yoruba | NHGRI |
| NA18498 | Africa | Yoruba | NHGRI |
| NA18499 | Africa | Yoruba | NHGRI |
| NA18501 | Africa | Yoruba | NHGRI |
| NA18502 | Africa | Yoruba | NHGRI |
| NA18505 | Africa | Yoruba | NHGRI |
| NA18508 | Africa | Yoruba | NHGRI |
| NA18510 | Africa | Yoruba | NHGRI |
| NA18511 | Africa | Yoruba | NHGRI |
| NA18517 | Africa | Yoruba | NHGRI |
| NA18519 | Africa | Yoruba | NHGRI |
| NA18520 | Africa | Yoruba | NHGRI |
| NA18523 | Africa | Yoruba | NHGRI |
| NA18852 | Africa | Yoruba | NHGRI |
| NA18855 | Africa | Yoruba | NHGRI |
| NA18858 | Africa | Yoruba | NHGRI |
| NA18861 | Africa | Yoruba | NHGRI |
| NA18867 | Africa | Yoruba | NHGRI |
| NA18868 | Africa | Yoruba | NHGRI |
| NA18870 | Africa | Yoruba | NHGRI |
| NA18873 | Africa | Yoruba | NHGRI |
| NA18874 | Africa | Yoruba | NHGRI |
| NA18907 | Africa | Yoruba | NHGRI |
| NA18908 | Africa | Yoruba | NHGRI |
| NA18909 | Africa | Yoruba | NHGRI |
| NA18910 | Africa | Yoruba | NHGRI |
| NA18912 | Africa | Yoruba | NHGRI |
| NA18916 | Africa | Yoruba | NHGRI |
| NA18917 | Africa | Yoruba | NHGRI |
| NA18923 | Africa | Yoruba | NHGRI |
| NA18924 | Africa | Yoruba | NHGRI |
| NA18933 | Africa | Yoruba | NHGRI |
| NA18934 | Africa | Yoruba | NHGRI |
| NA19027 | Africa | Luhya | NHGRI |
| NA19028 | Africa | Luhya | NHGRI |
| NA19031 | Africa | Luhya | NHGRI |
| NA19035 | Africa | Luhya | NHGRI |
| NA19038 | Africa | Luhya | NHGRI |
| NA19041 | Africa | Luhya | NHGRI |
| NA19044 | Africa | Luhya | NHGRI |
| NA19046 | Africa | Luhya | NHGRI |
| NA19093 | Africa | Yoruba | NHGRI |
| NA19095 | Africa | Yoruba | NHGRI |
| NA19096 | Africa | Yoruba | NHGRI |
| NA19099 | Africa | Yoruba | NHGRI |
| NA19102 | Africa | Yoruba | NHGRI |
| NA19107 | Africa | Yoruba | NHGRI |
| NA19108 | Africa | Yoruba | NHGRI |
| NA19113 | Africa | Yoruba | NHGRI |
| NA19114 | Africa | Yoruba | NHGRI |
| NA19116 | Africa | Yoruba | NHGRI |
| NA19117 | Africa | Yoruba | NHGRI |
| NA19121 | Africa | Yoruba | NHGRI |
| NA19122 | Africa | Yoruba | NHGRI |
| NA19127 | Africa | Yoruba | NHGRI |
| NA19131 | Africa | Yoruba | NHGRI |
| NA19137 | Africa | Yoruba | NHGRI |
| NA19140 | Africa | Yoruba | NHGRI |
| NA19143 | Africa | Yoruba | NHGRI |
| NA19146 | Africa | Yoruba | NHGRI |
| NA19147 | Africa | Yoruba | NHGRI |
| NA19149 | Africa | Yoruba | NHGRI |
| NA19150 | Africa | Yoruba | NHGRI |
| NA19152 | Africa | Yoruba | NHGRI |
| NA19159 | Africa | Yoruba | NHGRI |
| NA19172 | Africa | Yoruba | NHGRI |
| NA19175 | Africa | Yoruba | NHGRI |
| NA19176 | Africa | Yoruba | NHGRI |
| NA19178 | Africa | Yoruba | NHGRI |
| NA19179 | Africa | Yoruba | NHGRI |
| NA19181 | Africa | Yoruba | NHGRI |
| NA19182 | Africa | Yoruba | NHGRI |
| NA19184 | Africa | Yoruba | NHGRI |
| NA19185 | Africa | Yoruba | NHGRI |
| NA19189 | Africa | Yoruba | NHGRI |
| NA19190 | Africa | Yoruba | NHGRI |
| NA19193 | Africa | Yoruba | NHGRI |
| NA19197 | Africa | Yoruba | NHGRI |
| NA19198 | Africa | Yoruba | NHGRI |
| NA19201 | Africa | Yoruba | NHGRI |
| NA19204 | Africa | Yoruba | NHGRI |
| NA19213 | Africa | Yoruba | NHGRI |
| NA19214 | Africa | Yoruba | NHGRI |
| NA19222 | Africa | Yoruba | NHGRI |
| NA19225 | Africa | Yoruba | NHGRI |
| NA19226 | Africa | Yoruba | NHGRI |
| NA19235 | Africa | Yoruba | NHGRI |
| NA19236 | Africa | Yoruba | NHGRI |
| NA19238 | Africa | Yoruba | NHGRI |
| NA19247 | Africa | Yoruba | NHGRI |
| NA19248 | Africa | Yoruba | NHGRI |
| NA19256 | Africa | Yoruba | NHGRI |
| NA19257 | Africa | Yoruba | NHGRI |
| NA19307 | Africa | Luhya | NHGRI |
| NA19308 | Africa | Luhya | NHGRI |
| NA19309 | Africa | Luhya | NHGRI |
| NA19310 | Africa | Luhya | NHGRI |
| NA19311 | Africa | Luhya | NHGRI |
| NA19313 | Africa | Luhya | NHGRI |
| NA19314 | Africa | Luhya | NHGRI |
| NA19315 | Africa | Luhya | NHGRI |
| NA19316 | Africa | Luhya | NHGRI |
| NA19317 | Africa | Luhya | NHGRI |
| NA19318 | Africa | Luhya | NHGRI |
| NA19319 | Africa | Luhya | NHGRI |
| NA19321 | Africa | Luhya | NHGRI |
| NA19324 | Africa | Luhya | NHGRI |
| NA19327 | Africa | Luhya | NHGRI |
| NA19328 | Africa | Luhya | NHGRI |
| NA19332 | Africa | Luhya | NHGRI |
| NA19334 | Africa | Luhya | NHGRI |
| NA19346 | Africa | Luhya | NHGRI |
| NA19347 | Africa | Luhya | NHGRI |
| NA19350 | Africa | Luhya | NHGRI |
| NA19352 | Africa | Luhya | NHGRI |
| NA19359 | Africa | Luhya | NHGRI |
| NA19360 | Africa | Luhya | NHGRI |
| NA19371 | Africa | Luhya | NHGRI |
| NA19372 | Africa | Luhya | NHGRI |
| NA19373 | Africa | Luhya | NHGRI |
| NA19374 | Africa | Luhya | NHGRI |
| NA19375 | Africa | Luhya | NHGRI |
| NA19376 | Africa | Luhya | NHGRI |
| NA19377 | Africa | Luhya | NHGRI |
| NA19379 | Africa | Luhya | NHGRI |
| NA19380 | Africa | Luhya | NHGRI |
| NA19381 | Africa | Luhya | NHGRI |
| NA19382 | Africa | Luhya | NHGRI |
| NA19383 | Africa | Luhya | NHGRI |
| NA19384 | Africa | Luhya | NHGRI |
| NA19385 | Africa | Luhya | NHGRI |
| NA19390 | Africa | Luhya | NHGRI |
| NA19391 | Africa | Luhya | NHGRI |
| NA19393 | Africa | Luhya | NHGRI |
| NA19394 | Africa | Luhya | NHGRI |
| NA19396 | Africa | Luhya | NHGRI |
| NA19397 | Africa | Luhya | NHGRI |
| NA19398 | Africa | Luhya | NHGRI |
| NA19399 | Africa | Luhya | NHGRI |
| NA19403 | Africa | Luhya | NHGRI |
| NA19404 | Africa | Luhya | NHGRI |
| NA19428 | Africa | Luhya | NHGRI |
| NA19429 | Africa | Luhya | NHGRI |
| NA19430 | Africa | Luhya | NHGRI |
| NA19431 | Africa | Luhya | NHGRI |
| NA19434 | Africa | Luhya | NHGRI |
| NA19435 | Africa | Luhya | NHGRI |
| NA19436 | Africa | Luhya | NHGRI |
| NA19437 | Africa | Luhya | NHGRI |
| NA19438 | Africa | Luhya | NHGRI |
| NA19439 | Africa | Luhya | NHGRI |
| NA19440 | Africa | Luhya | NHGRI |
| NA19443 | Africa | Luhya | NHGRI |
| NA19444 | Africa | Luhya | NHGRI |
| NA19445 | Africa | Luhya | NHGRI |
| NA19446 | Africa | Luhya | NHGRI |
| NA19448 | Africa | Luhya | NHGRI |
| NA19449 | Africa | Luhya | NHGRI |
| NA19451 | Africa | Luhya | NHGRI |
| NA19452 | Africa | Luhya | NHGRI |
| NA19455 | Africa | Luhya | NHGRI |
| NA19456 | Africa | Luhya | NHGRI |
| NA19457 | Africa | Luhya | NHGRI |
| NA19462 | Africa | Luhya | NHGRI |
| NA19463 | Africa | Luhya | NHGRI |
| NA19466 | Africa | Luhya | NHGRI |
| NA19467 | Africa | Luhya | NHGRI |
| NA19468 | Africa | Luhya | NHGRI |
| NA19469 | Africa | Luhya | NHGRI |
| NA19470 | Africa | Luhya | NHGRI |
| NA19471 | Africa | Luhya | NHGRI |
| NA19472 | Africa | Luhya | NHGRI |
| NA19473 | Africa | Luhya | NHGRI |
| NA19474 | Africa | Luhya | NHGRI |
| NA18524 | East Asia | Han Chinese | NHGRI |
| NA18526 | East Asia | Han Chinese | NHGRI |
| NA18529 | East Asia | Han Chinese | NHGRI |
| NA18532 | East Asia | Han Chinese | NHGRI |
| NA18537 | East Asia | Han Chinese | NHGRI |
| NA18540 | East Asia | Han Chinese | NHGRI |
| NA18542 | East Asia | Han Chinese | NHGRI |
| NA18545 | East Asia | Han Chinese | NHGRI |
| NA18547 | East Asia | Han Chinese | NHGRI |
| NA18550 | East Asia | Han Chinese | NHGRI |
| NA18552 | East Asia | Han Chinese | NHGRI |
| NA18555 | East Asia | Han Chinese | NHGRI |
| NA18558 | East Asia | Han Chinese | NHGRI |
| NA18561 | East Asia | Han Chinese | NHGRI |
| NA18562 | East Asia | Han Chinese | NHGRI |
| NA18563 | East Asia | Han Chinese | NHGRI |
| NA18564 | East Asia | Han Chinese | NHGRI |
| NA18566 | East Asia | Han Chinese | NHGRI |
| NA18570 | East Asia | Han Chinese | NHGRI |
| NA18571 | East Asia | Han Chinese | NHGRI |
| NA18572 | East Asia | Han Chinese | NHGRI |
| NA18573 | East Asia | Han Chinese | NHGRI |
| NA18576 | East Asia | Han Chinese | NHGRI |
| NA18577 | East Asia | Han Chinese | NHGRI |
| NA18579 | East Asia | Han Chinese | NHGRI |
| NA18582 | East Asia | Han Chinese | NHGRI |
| NA18592 | East Asia | Han Chinese | NHGRI |
| NA18593 | East Asia | Han Chinese | NHGRI |
| NA18594 | East Asia | Han Chinese | NHGRI |
| NA18603 | East Asia | Han Chinese | NHGRI |
| NA18605 | East Asia | Han Chinese | NHGRI |
| NA18608 | East Asia | Han Chinese | NHGRI |
| NA18609 | East Asia | Han Chinese | NHGRI |
| NA18611 | East Asia | Han Chinese | NHGRI |
| NA18612 | East Asia | Han Chinese | NHGRI |
| NA18620 | East Asia | Han Chinese | NHGRI |
| NA18621 | East Asia | Han Chinese | NHGRI |
| NA18622 | East Asia | Han Chinese | NHGRI |
| NA18623 | East Asia | Han Chinese | NHGRI |
| NA18624 | East Asia | Han Chinese | NHGRI |
| NA18632 | East Asia | Han Chinese | NHGRI |
| NA18633 | East Asia | Han Chinese | NHGRI |
| NA18635 | East Asia | Han Chinese | NHGRI |
| NA18636 | East Asia | Han Chinese | NHGRI |
| NA18637 | East Asia | Han Chinese | NHGRI |
| NA18940 | East Asia | Japanese | NHGRI |
| NA18942 | East Asia | Japanese | NHGRI |
| NA18943 | East Asia | Japanese | NHGRI |
| NA18944 | East Asia | Japanese | NHGRI |
| NA18945 | East Asia | Japanese | NHGRI |
| NA18947 | East Asia | Japanese | NHGRI |
| NA18948 | East Asia | Japanese | NHGRI |
| NA18949 | East Asia | Japanese | NHGRI |
| NA18951 | East Asia | Japanese | NHGRI |
| NA18952 | East Asia | Japanese | NHGRI |
| NA18953 | East Asia | Japanese | NHGRI |
| NA18956 | East Asia | Japanese | NHGRI |
| NA18959 | East Asia | Japanese | NHGRI |
| NA18960 | East Asia | Japanese | NHGRI |
| NA18961 | East Asia | Japanese | NHGRI |
| NA18964 | East Asia | Japanese | NHGRI |
| NA18965 | East Asia | Japanese | NHGRI |
| NA18966 | East Asia | Japanese | NHGRI |
| NA18967 | East Asia | Japanese | NHGRI |
| NA18968 | East Asia | Japanese | NHGRI |
| NA18969 | East Asia | Japanese | NHGRI |
| NA18970 | East Asia | Japanese | NHGRI |
| NA18971 | East Asia | Japanese | NHGRI |
| NA18972 | East Asia | Japanese | NHGRI |
| NA18973 | East Asia | Japanese | NHGRI |
| NA18974 | East Asia | Japanese | NHGRI |
| NA18975 | East Asia | Japanese | NHGRI |
| NA18976 | East Asia | Japanese | NHGRI |
| NA18978 | East Asia | Japanese | NHGRI |
| NA18980 | East Asia | Japanese | NHGRI |
| NA18981 | East Asia | Japanese | NHGRI |
| NA18987 | East Asia | Japanese | NHGRI |
| NA18990 | East Asia | Japanese | NHGRI |
| NA18991 | East Asia | Japanese | NHGRI |
| NA18992 | East Asia | Japanese | NHGRI |
| NA18994 | East Asia | Japanese | NHGRI |
| NA18995 | East Asia | Japanese | NHGRI |
| NA18997 | East Asia | Japanese | NHGRI |
| NA18998 | East Asia | Japanese | NHGRI |
| NA18999 | East Asia | Japanese | NHGRI |
| NA19000 | East Asia | Japanese | NHGRI |
| NA19003 | East Asia | Japanese | NHGRI |
| NA19005 | East Asia | Japanese | NHGRI |
| NA19012 | East Asia | Japanese | NHGRI |
| ND00717 | Caucasian | Caucasian | NINDS |
| ND01087 | Caucasian | Caucasian | NINDS |
| ND01254 | Caucasian | Caucasian | NINDS |
| ND01606 | Caucasian | Caucasian | NINDS |
| ND01740 | Caucasian | Caucasian | NINDS |
| ND01756 | Caucasian | Caucasian | NINDS |
| ND02381 | Caucasian | Caucasian | NINDS |
| ND02532 | Caucasian | Caucasian | NINDS |
| ND03376 | Caucasian | Caucasian | NINDS |
| ND03967 | Caucasian | Caucasian | NINDS |
| ND03968 | Caucasian | Caucasian | NINDS |
| ND04113 | Caucasian | Caucasian | NINDS |
| ND04117 | Caucasian | Caucasian | NINDS |
| ND04311 | Caucasian | Caucasian | NINDS |
| ND04313 | Caucasian | Caucasian | NINDS |
| ND04337 | Caucasian | Caucasian | NINDS |
| ND04402 | Caucasian | Caucasian | NINDS |
| ND04632 | Caucasian | Caucasian | NINDS |
| ND04990 | Caucasian | Caucasian | NINDS |
| ND05123 | Caucasian | Caucasian | NINDS |
| ND05211 | Caucasian | Caucasian | NINDS |
| ND05438 | Caucasian | Caucasian | NINDS |
| ND05439 | Caucasian | Caucasian | NINDS |
| ND05706 | Caucasian | Caucasian | NINDS |
| ND05873 | Caucasian | Caucasian | NINDS |
| ND06140 | Caucasian | Caucasian | NINDS |
| ND06141 | Caucasian | Caucasian | NINDS |
| ND06166 | Caucasian | Caucasian | NINDS |
| ND06247 | Caucasian | Caucasian | NINDS |
| ND06501 | Caucasian | Caucasian | NINDS |
| ND07601 | Caucasian | Caucasian | NINDS |
| ND07944 | Caucasian | Caucasian | NINDS |
| ND07955 | Caucasian | Caucasian | NINDS |
| ND08965 | Caucasian | Caucasian | NINDS |
| ND08966 | Caucasian | Caucasian | NINDS |
| ND08984 | Caucasian | Caucasian | NINDS |
| ND08995 | Caucasian | Caucasian | NINDS |
| ND09211 | Caucasian | Caucasian | NINDS |
| ND09219 | Caucasian | Caucasian | NINDS |
| ND09323 | Caucasian | Caucasian | NINDS |
| ND09349 | Caucasian | Caucasian | NINDS |
| ND09400 | Caucasian | Caucasian | NINDS |
| ND09419 | Caucasian | Caucasian | NINDS |
| ND09424 | Caucasian | Caucasian | NINDS |
| ND09456 | Caucasian | Caucasian | NINDS |
| ND09458 | Caucasian | Caucasian | NINDS |
| ND09485 | Caucasian | Caucasian | NINDS |
| ND09521 | Caucasian | Caucasian | NINDS |
| ND09609 | Caucasian | Caucasian | NINDS |
| ND09668 | Caucasian | Caucasian | NINDS |
| ND09686 | Caucasian | Caucasian | NINDS |
| ND09882 | Caucasian | Caucasian | NINDS |
| ND09892 | Caucasian | Caucasian | NINDS |
| ND09908 | Caucasian | Caucasian | NINDS |
| ND09912 | Caucasian | Caucasian | NINDS |
| ND09941 | Caucasian | Caucasian | NINDS |
| ND09958 | Caucasian | Caucasian | NINDS |
| ND09991 | Caucasian | Caucasian | NINDS |
| ND10008 | Caucasian | Caucasian | NINDS |
| ND10017 | Caucasian | Caucasian | NINDS |
| ND10039 | Caucasian | Caucasian | NINDS |
| ND10120 | Caucasian | Caucasian | NINDS |
| ND10271 | Caucasian | Caucasian | NINDS |
| ND10278 | Caucasian | Caucasian | NINDS |
| ND10279 | Caucasian | Caucasian | NINDS |
| ND10305 | Caucasian | Caucasian | NINDS |
| ND10325 | Caucasian | Caucasian | NINDS |
| ND10344 | Caucasian | Caucasian | NINDS |
| ND10364 | Caucasian | Caucasian | NINDS |
| ND10389 | Caucasian | Caucasian | NINDS |
| ND10423 | Caucasian | Caucasian | NINDS |
| ND10428 | Caucasian | Caucasian | NINDS |
| ND10429 | Caucasian | Caucasian | NINDS |
| ND10471 | Caucasian | Caucasian | NINDS |
| ND10474 | Caucasian | Caucasian | NINDS |
| ND10480 | Caucasian | Caucasian | NINDS |
| ND10499 | Caucasian | Caucasian | NINDS |
| ND10525 | Caucasian | Caucasian | NINDS |
| ND10540 | Caucasian | Caucasian | NINDS |
| ND10560 | Caucasian | Caucasian | NINDS |
| ND10680 | Caucasian | Caucasian | NINDS |
| ND10712 | Caucasian | Caucasian | NINDS |
| ND10726 | Caucasian | Caucasian | NINDS |
| ND10878 | Caucasian | Caucasian | NINDS |
| ND10881 | Caucasian | Caucasian | NINDS |
| ND10883 | Caucasian | Caucasian | NINDS |
| ND10886 | Caucasian | Caucasian | NINDS |
| ND10887 | Caucasian | Caucasian | NINDS |
| ND10888 | Caucasian | Caucasian | NINDS |
| ND10891 | Caucasian | Caucasian | NINDS |
| ND10970 | Caucasian | Caucasian | NINDS |
| ND10980 | Caucasian | Caucasian | NINDS |
| ND00485 | African American | African American | NINDS |
| ND00662 | African American | African American | NINDS |
| ND03137 | African American | African American | NINDS |
| ND03973 | African American | African American | NINDS |
| ND04009 | African American | African American | NINDS |
| ND04010 | African American | African American | NINDS |
| ND04011 | African American | African American | NINDS |
| ND04013 | African American | African American | NINDS |
| ND04020 | African American | African American | NINDS |
| ND04030 | African American | African American | NINDS |
| ND04385 | African American | African American | NINDS |
| ND04478 | African American | African American | NINDS |
| ND04479 | African American | African American | NINDS |
| ND04480 | African American | African American | NINDS |
| ND04691 | African American | African American | NINDS |
| ND04971 | African American | African American | NINDS |
| ND04978 | African American | African American | NINDS |
| ND05016 | African American | African American | NINDS |
| ND05233 | African American | African American | NINDS |
| ND05278 | African American | African American | NINDS |
| ND05601 | African American | African American | NINDS |
| ND07547 | African American | African American | NINDS |
| ND08239 | African American | African American | NINDS |
| ND08253 | African American | African American | NINDS |
| ND08502 | African American | African American | NINDS |
| ND09401 | African American | African American | NINDS |
| ND09402 | African American | African American | NINDS |
| ND09523 | African American | African American | NINDS |
| ND09555 | African American | African American | NINDS |
| ND09611 | African American | African American | NINDS |
| ND09658 | African American | African American | NINDS |
| ND09688 | African American | African American | NINDS |
| ND09723 | African American | African American | NINDS |
| ND09724 | African American | African American | NINDS |
| ND09725 | African American | African American | NINDS |
| ND09726 | African American | African American | NINDS |
| ND09728 | African American | African American | NINDS |
| ND09732 | African American | African American | NINDS |
| ND09790 | African American | African American | NINDS |
| ND09824 | African American | African American | NINDS |
| ND09825 | African American | African American | NINDS |
| ND09828 | African American | African American | NINDS |
| ND09938 | African American | African American | NINDS |
| ND09947 | African American | African American | NINDS |
| ND09951 | African American | African American | NINDS |
| ND09952 | African American | African American | NINDS |
| ND09967 | African American | African American | NINDS |
| ND10128 | African American | African American | NINDS |
| ND10331 | African American | African American | NINDS |
| ND10348 | African American | African American | NINDS |
| ND10359 | African American | African American | NINDS |
| ND10633 | African American | African American | NINDS |
| ND10856 | African American | African American | NINDS |
| ND11002 | African American | African American | NINDS |
| ND11082 | African American | African American | NINDS |
| ND11086 | African American | African American | NINDS |
| ND11300 | African American | African American | NINDS |
| ND11328 | African American | African American | NINDS |
| ND11329 | African American | African American | NINDS |
| ND11331 | African American | African American | NINDS |
| ND11332 | African American | African American | NINDS |
| ND11333 | African American | African American | NINDS |
| ND11414 | African American | African American | NINDS |
| ND11559 | African American | African American | NINDS |
| ND11656 | African American | African American | NINDS |
| ND11905 | African American | African American | NINDS |
| ND11906 | African American | African American | NINDS |
| ND11908 | African American | African American | NINDS |
| ND12118 | African American | African American | NINDS |
| ND12143 | African American | African American | NINDS |
| ND12147 | African American | African American | NINDS |
| ND12149 | African American | African American | NINDS |
| ND12151 | African American | African American | NINDS |
| ND12280 | African American | African American | NINDS |
| ND12386 | African American | African American | NINDS |
| ND12395 | African American | African American | NINDS |
| ND12571 | African American | African American | NINDS |
| ND12969 | African American | African American | NINDS |
| ND13117 | African American | African American | NINDS |
| ND13434 | African American | African American | NINDS |
| ND13554 | African American | African American | NINDS |
| ND13784 | African American | African American | NINDS |
| ND13927 | African American | African American | NINDS |
| ND13935 | African American | African American | NINDS |
| ND14116 | African American | African American | NINDS |
| ND14118 | African American | African American | NINDS |
| ND14336 | African American | African American | NINDS |
| ND14430 | African American | African American | NINDS |
| ND14431 | African American | African American | NINDS |
| ND14432 | African American | African American | NINDS |
| ND14711 | African American | African American | NINDS |
| ND14835 | African American | African American | NINDS |
| ND02669 | Hispanic | Hispanic | NINDS |
| ND04295 | Hispanic | Hispanic | NINDS |
| ND06919 | Hispanic | Hispanic | NINDS |
| ND08582 | Hispanic | Hispanic | NINDS |
| ND11653 | Hispanic | Hispanic | NINDS |
| ND12207 | Hispanic | Hispanic | NINDS |
| ND12208 | Hispanic | Hispanic | NINDS |
| ND12415 | Hispanic | Hispanic | NINDS |
| ND12499 | Hispanic | Hispanic | NINDS |
| ND12536 | Hispanic | Hispanic | NINDS |
| ND12539 | Hispanic | Hispanic | NINDS |
| ND12698 | Hispanic | Hispanic | NINDS |
| ND12715 | Hispanic | Hispanic | NINDS |
| ND12784 | Hispanic | Hispanic | NINDS |
| ND13111 | Hispanic | Hispanic | NINDS |
| ND13388 | Hispanic | Hispanic | NINDS |
| ND13390 | Hispanic | Hispanic | NINDS |
| ND13430 | Hispanic | Hispanic | NINDS |
| ND13885 | Hispanic | Hispanic | NINDS |
| ND13887 | Hispanic | Hispanic | NINDS |
| ND13992 | Hispanic | Hispanic | NINDS |
| ND14229 | Hispanic | Hispanic | NINDS |
| ND14279 | Hispanic | Hispanic | NINDS |
| ND14632 | Hispanic | Hispanic | NINDS |
| ND14808 | Hispanic | Hispanic | NINDS |
| ND14813 | Hispanic | Hispanic | NINDS |
| ND14849 | Hispanic | Hispanic | NINDS |
| ND14896 | Hispanic | Hispanic | NINDS |
| ND15032 | Hispanic | Hispanic | NINDS |
| ND15223 | Hispanic | Hispanic | NINDS |
| ND15522 | Hispanic | Hispanic | NINDS |
| ND15649 | Hispanic | Hispanic | NINDS |
| ND15728 | Hispanic | Hispanic | NINDS |
| ND15757 | Hispanic | Hispanic | NINDS |
| ND15786 | Hispanic | Hispanic | NINDS |
| ND15787 | Hispanic | Hispanic | NINDS |
| ND15810 | Hispanic | Hispanic | NINDS |
| ND15850 | Hispanic | Hispanic | NINDS |
| ND15851 | Hispanic | Hispanic | NINDS |
| ND15926 | Hispanic | Hispanic | NINDS |
| ND16030 | Hispanic | Hispanic | NINDS |
| ND16076 | Hispanic | Hispanic | NINDS |
| ND16077 | Hispanic | Hispanic | NINDS |
| ND16081 | Hispanic | Hispanic | NINDS |
| ND16237 | Hispanic | Hispanic | NINDS |
| ND16245 | Hispanic | Hispanic | NINDS |
| ND16350 | Hispanic | Hispanic | NINDS |
| ND16351 | Hispanic | Hispanic | NINDS |
| ND16535 | Hispanic | Hispanic | NINDS |
| ND16536 | Hispanic | Hispanic | NINDS |
| ND16564 | Hispanic | Hispanic | NINDS |
| ND16565 | Hispanic | Hispanic | NINDS |
| ND16599 | Hispanic | Hispanic | NINDS |
| ND16602 | Hispanic | Hispanic | NINDS |
| ND16636 | Hispanic | Hispanic | NINDS |
| ND16669 | Hispanic | Hispanic | NINDS |
| ND19238 | Hispanic | Hispanic | NINDS |
| ND19240 | Hispanic | Hispanic | NINDS |
| ND19330 | Hispanic | Hispanic | NINDS |
| ND19488 | Hispanic | Hispanic | NINDS |
| ND19489 | Hispanic | Hispanic | NINDS |
| ND19531 | Hispanic | Hispanic | NINDS |
| ND19738 | Hispanic | Hispanic | NINDS |
| ND19740 | Hispanic | Hispanic | NINDS |
| ND19792 | Hispanic | Hispanic | NINDS |
| ND19797 | Hispanic | Hispanic | NINDS |
| ND19922 | Hispanic | Hispanic | NINDS |
| ND19981 | Hispanic | Hispanic | NINDS |
| ND19982 | Hispanic | Hispanic | NINDS |
| ND19985 | Hispanic | Hispanic | NINDS |
| ND20030 | Hispanic | Hispanic | NINDS |
| ND20031 | Hispanic | Hispanic | NINDS |
| ND20197 | Hispanic | Hispanic | NINDS |
| ND20279 | Hispanic | Hispanic | NINDS |
| ND20394 | Hispanic | Hispanic | NINDS |
| ND20397 | Hispanic | Hispanic | NINDS |
| ND20398 | Hispanic | Hispanic | NINDS |
| ND20399 | Hispanic | Hispanic | NINDS |
| ND20515 | Hispanic | Hispanic | NINDS |
| ND20627 | Hispanic | Hispanic | NINDS |
| ND21543 | Hispanic | Hispanic | NINDS |
| ND21917 | Hispanic | Hispanic | NINDS |
| ND21922 | Hispanic | Hispanic | NINDS |
| ND21925 | Hispanic | Hispanic | NINDS |
| ND22006 | Hispanic | Hispanic | NINDS |
| ND22007 | Hispanic | Hispanic | NINDS |
| ND22026 | Hispanic | Hispanic | NINDS |
| ND22142 | Hispanic | Hispanic | NINDS |
| ND22143 | Hispanic | Hispanic | NINDS |
| ND22553 | Hispanic | Hispanic | NINDS |
| ND22732 | Hispanic | Hispanic | NINDS |
| ND22734 | Hispanic | Hispanic | NINDS |
| ND06942 | Non-Caucasian Hispanic | Non-Caucasian Hispanic | NINDS |
| ND10342 | Non-Caucasian Hispanic | Non-Caucasian Hispanic | NINDS |
| ND10884 | Non-Caucasian Hispanic | Non-Caucasian Hispanic | NINDS |
| ND23420 | Non-Caucasian Hispanic | Non-Caucasian Hispanic | NINDS |
| ND24027 | Non-Caucasian Hispanic | Non-Caucasian Hispanic | NINDS |
| ND24033 | Non-Caucasian Hispanic | Non-Caucasian Hispanic | NINDS |
| ND24035 | Non-Caucasian Hispanic | Non-Caucasian Hispanic | NINDS |
| ND24036 | Non-Caucasian Hispanic | Non-Caucasian Hispanic | NINDS |
| ND24038 | Non-Caucasian Hispanic | Non-Caucasian Hispanic | NINDS |
| ND24039 | Non-Caucasian Hispanic | Non-Caucasian Hispanic | NINDS |
| ND24060 | Non-Caucasian Hispanic | Non-Caucasian Hispanic | NINDS |
| ND24201 | Non-Caucasian Hispanic | Non-Caucasian Hispanic | NINDS |
| ND24278 | Non-Caucasian Hispanic | Non-Caucasian Hispanic | NINDS |
| ND24341 | Non-Caucasian Hispanic | Non-Caucasian Hispanic | NINDS |
| ND24387 | Non-Caucasian Hispanic | Non-Caucasian Hispanic | NINDS |
| ND24390 | Non-Caucasian Hispanic | Non-Caucasian Hispanic | NINDS |
| ND24554 | Non-Caucasian Hispanic | Non-Caucasian Hispanic | NINDS |
| ND06150 | Asian | Asian | NINDS |
| ND06515 | Asian | Asian | NINDS |
| ND07692 | Asian | Asian | NINDS |
| ND08934 | Asian | Asian | NINDS |
| ND09347 | Asian | Asian | NINDS |
| ND09399 | Asian | Asian | NINDS |
| ND09505 | Asian | Asian | NINDS |
| ND09619 | Asian | Asian | NINDS |
| ND09894 | Asian | Asian | NINDS |
| ND09895 | Asian | Asian | NINDS |
| ND10102 | Asian | Asian | NINDS |
| ND10193 | Asian | Asian | NINDS |
| ND10300 | Asian | Asian | NINDS |
| ND10333 | Asian | Asian | NINDS |
| ND10361 | Asian | Asian | NINDS |
| ND10363 | Asian | Asian | NINDS |
| ND10381 | Asian | Asian | NINDS |
| ND10426 | Asian | Asian | NINDS |
| ND10431 | Asian | Asian | NINDS |
| ND00293 | American Indian | American Indian | NINDS |
| ND01188 | American Indian | American Indian | NINDS |
| ND02442 | American Indian | American Indian | NINDS |
| ND04588 | American Indian | American Indian | NINDS |
| ND04589 | American Indian | American Indian | NINDS |
| ND04720 | American Indian | American Indian | NINDS |
| ND04913 | American Indian | American Indian | NINDS |
| ND06119 | American Indian | American Indian | NINDS |
| ND06137 | American Indian | American Indian | NINDS |
| ND07533 | American Indian | American Indian | NINDS |
| ND08797 | American Indian | American Indian | NINDS |
| ND09082 | American Indian | American Indian | NINDS |
| ND24208 | American Indian | American Indian | NINDS |
| ND01405 | Pacific Islander | Pacific Islander | NINDS |
| ND03636 | Pacific Islander | Pacific Islander | NINDS |
| ND05266 | Pacific Islander | Pacific Islander | NINDS |
| ND06630 | Pacific Islander | Pacific Islander | NINDS |
| ND09942 | Pacific Islander | Pacific Islander | NINDS |
| ND10873 | Pacific Islander | Pacific Islander | NINDS |
| ND12356 | Pacific Islander | Pacific Islander | NINDS |
| ND13334 | Pacific Islander | Pacific Islander | NINDS |
| ND15508 | Pacific Islander | Pacific Islander | NINDS |
| ND15573 | Pacific Islander | Pacific Islander | NINDS |
| ND15574 | Pacific Islander | Pacific Islander | NINDS |
| ND20135 | Pacific Islander | Pacific Islander | NINDS |
| ND09807 | More than one race | More than one race | NINDS |
| ND10293 | More than one race | More than one race | NINDS |
| ND13099 | More than one race | More than one race | NINDS |
| ND13116 | More than one race | More than one race | NINDS |
| ND13658 | More than one race | More than one race | NINDS |
| ND13918 | More than one race | More than one race | NINDS |
| ND14148 | More than one race | More than one race | NINDS |
| ND14918 | More than one race | More than one race | NINDS |
| ND14966 | More than one race | More than one race | NINDS |
| ND15000 | More than one race | More than one race | NINDS |
| ND08351 | Unknown | Unknown | NINDS |
| ND09099 | Unknown | Unknown | NINDS |
| ND09817 | Unknown | Unknown | NINDS |
| ND09896 | Unknown | Unknown | NINDS |
| ND13624 | Unknown | Unknown | NINDS |
| ND14024 | Unknown | Unknown | NINDS |
| ND14051 | Unknown | Unknown | NINDS |
| ND14236 | Unknown | Unknown | NINDS |
| ND14939 | Unknown | Unknown | NINDS |
| ND14950 | Unknown | Unknown | NINDS |
| ND24643 | Unknown | Unknown | NINDS |
| ND24687 | Unknown | Unknown | NINDS |
| ND24980 | Unknown | Unknown | NINDS |
| ND24996 | Unknown | Unknown | NINDS |
| ND25008 | Unknown | Unknown | NINDS |
| ND25010 | Unknown | Unknown | NINDS |
| ND25070 | Unknown | Unknown | NINDS |
| ND25206 | Unknown | Unknown | NINDS |
| ND25207 | Unknown | Unknown | NINDS |
| ND25250 | Unknown | Unknown | NINDS |

^*^Samples from the NINDS Repository are all of self-declared race
