# Supplementary material for: CoAIMs: A Cost-Effective Panel of Ancestry Informative Markers for Determining Continental Origins
Source: PLoS One. 2010 Oct 15;5(10):e13443. doi: 10.1371/journal.pone.0013443 (PMC2955551; doi:10.1371/journal.pone.0013443)
Supplement: Table S2 — The 19 MSATs of the ABI Identifiler and Coriell Identity Mapping kit and their Fst values. (0.04 MB DOC) [file pone.0013443.s005.doc]

| **MSAT** | **Panel** | **Fst** |
| --- | --- | --- |
| D2S1338 | ABI Identifiler | 0.047 |
| D18S51 | ABI Identifiler | 0.030 |
| D19S433 | ABI Identifiler | 0.031 |
| D13S317 | ABI Identifiler | 0.053 |
| D21S11 | ABI Identifiler | 0.035 |
| FGA | ABI Identifiler | 0.028 |
| D5S818 | ABI Identifiler | 0.053 |
| TPOX | ABI Identifiler | 0.047 |
| D8S1179 | ABI Identifiler | 0.030 |
| D16S539 | ABI Identifiler | 0.022 |
| D3S1358 | ABI Identifiler | 0.039 |
| D7S820 | ABI Identifiler | 0.028 |
| CSF1PO | ABI Identifiler | 0.015 |
| TH01* | ABI Identifiler/Coriell Identity Mapping Set | 0.063 |
| VWA31* | ABI Identifiler/Coriell Identity Mapping Set | 0.025 |
| D22S417 | Coriell Identity Mapping Set | 0.049 |
| D10S526 | Coriell Identity Mapping Set | 0.019 |
| D5S592 | Coriell Identity Mapping Set | 0.037 |
| FES/FPS | Coriell Identity Mapping Set | 0.038 |
|  | **Average** | **0.036 ± 0.013** |
|  | *THO1 and VWA31 are in both panels | |
