# Supplementary material for: CoAIMs: A Cost-Effective Panel of Ancestry Informative Markers for Determining Continental Origins
Source: PLoS One. 2010 Oct 15;5(10):e13443. doi: 10.1371/journal.pone.0013443 (PMC2955551; doi:10.1371/journal.pone.0013443)
Supplement: Table S3 — MSATs of the CoAIM panel. (0.02 MB DOCX) [file pone.0013443.s006.docx]

| **MSAT** | **Chr** | **Chomosomal region*** | **Repeat number** | **BP range** | **Panel** | **F_st_ Value** | **Forward Primer^†^** | **Reverse Primer^†^** | **primer conc. (uM)** |
| --- | --- | --- | --- | --- | --- | --- | --- | --- | --- |
| D1S1589 | 1 | 172527725-172527928 | 3 | 190-223 | 1 | 0.167 | TACTCAGGAGGCAGAGATGG | PET-CTGCTTTGGGTTTCACTTGT | 0.3 |
| TTTA063P | 1 | 16309367-16309581 | 2 | 185-214 | 1 | 0.211 | GTTTCTTAGCTGAGATCCCACCACTTG | NED-AATCCATGGCTGCTCCAGT | 0.3 |
| D2S2986 | 2 | 242515162-242515311 | 2 | 148-166 | 1 | 0.164 | NED-GTGAGGTCAGGAACGCTAAG | CTGTCATCGCTATGAGCCAG | 0.05 |
| D3S1311 | 3 | 198502504-198502647 | 2 | 122-158 | 1 | 0.142 | 6FAM-GGAAGTTTCAGCCAACG | TTAGTCCCACTGATGTTACATTT | 0.6 |
| ATT015 | 4 | 13535740-13536033 | 2 | 270-301 | 1 | 0.148 | AGCACCCATATTTGCAGGAA | 6FAM-TCACACTCTGGGGACTGTTG | 0.8 |
| D5S1501 | 5 | 78504210-78504318 | 2 | 82-122 | 1 | 0.110 | TTATAGAGCAGCCTTCCCTG | PET-AAAAGCCTCCAGTGGGATAT | 0.4 |
| D8S560 | 8 | 21646249-21646398 | 2 | 132-176 | 1 | 0.193 | PET-GGCATTTCAGAGGACC | TGCAAAGATGGGCTCAG | 0.8 |
| D9S1779 | 9 | 433870-433992 | 2 | 116-156 | 1 | 0.217 | VIC-CCCTGCCAGGTGTGCATCTG | TCAGGCTCCCTCGTGGCTCT | 0.05 |
| D11S935 | 11 | 35979755-35980038 | 2 | 187-211 | 1 | 0.160 | VIC-TACTAACCAAAAGAGTTGGGG | CTATCATTCAGAAAATGTTGGC | 0.3 |
| GTT035 | 13 | 113070528-113070772 | 2 | 239-270 | 1 | 0.136 | VIC-ACAGGGGTCAATCACTGGAG | AAAATTAGCCAGGCATGGTG | 0.2 |
| D16S422 | 16 | 81468865-81469015 | 2 | 178-224 | 1 | 0.170 | 6FAM-CAGTGTAACCTGGGGGC | CTTTCGATTAGTTTAGCAGAATGAG | 1.1 |
| TTA015P | 22 | 21943754-21943884 | 2 | 108-138 | 1 | 0.174 | NED-ACTGGAAAGTGCTGCATGTG | GTTTCTTCTGGGCAGCAGAGTGAAACT | 0.05 |
|  |  |  |  |  |  |  |  |  |  |
| D1S534 | 1 | 119479787-119479989 | 2 | 188-224 | 2 | 0.117 | PET-AGCACATAGCAGGCACTAGC | CGATTGTGCCACTACACAGT | 0.2 |
| D2S441 | 2 | 689092502-68092661 | 2 | 127-159 | 2 | 0.132 | VIC-ATTGGAGCTAAGTGGCTGTG | AAAAGGCTGTAACAAGGGCT | 0.08 |
| D3S1262 | 3 | 187706173-187706288 | 2 | 102-130 | 2 | 0.135 | CGGCCCTAGGATATTTTCAA | 6FAM-CCAGTTTTTATGGACGGGGT | 0.7 |
| D3S1583 | 3 | 25530112-25530260 | 2 | 149-173 | 2 | 0.252 | AGCTTGTAAATAGGTCCTAACAGAG | NED-TGGTTTAATAGGCACCGTTT | 0.3 |
| D6S1037 | 6 | 118891794-118892113 | 2 | 317-329 | 2 | 0.203 | PET-TCCTTCCTCACTGTTTGAGC | CAGAATGAATAGGAATGTGGC | 0.7 |
| AAT095 | 17 | 76215054-76215313 | 2 | 249-281 | 2 | 0.125 | TTGGGATGGTGAAAAGGTTC | PET-TGCTCACGGTTGTCCTGTAG | 0.4 |
| D9S1871 | 9 | 3849269-3849443 | 2 | 143-179 | 2 | 0.153 | 6FAM-ACTATTGTTGGAGGCAGG | TTAACTTTTGGGGACAGAA | 1.4 |
| D9S1777 | 9 | 70377475-70377715 | 2 | 235-247 | 2 | 0.190 | VIC-AGGCAATGCTGGTTCATAG | CTCCCAAATACCTGTTACCC | 0.2 |
| D11S2000 | 11 | 105063951-105064165 | 2 | 193-241 | 2 | 0.117 | NED-AGTAGAGAACAAAACACTGTGGC | TTTGAAGATCTGTGAAATGTGC | 0.2 |
| TTTA028 | 16 | 606309-606487 | 4 | 183-207 | 2 | 0.179 | VIC-CAGTCCAGAGTCCGGAGAAG | GGCACCTTTAATCCCAGCTA | 0.4 |
| AAT083 | 17 | 17054235-17054532 | 2 | 259-319 | 2 | 0.175 | 6FAM-GTTTTGGAAACCTCCGGACT | ACTGCAGGCTCGAAATCCTA | 0.4 |
| D22S1169 | 22 | 47788105-47788230 | 2 | 114-136 | 2 | 0.180 | GCACACACATGCACATAATC | NED-AACAACTTCCAGCAGACG | 0.2 |
|  |  |  |  |  |  |  |  |  |  |
| D1S1622 | 1 | 30083541-30083800 | 2 | 256-283 | 3 | 0.150 | CCCTCTGTCTCCAGCTGTAA | 6FAM-TCACCCTCACATGATGCC | 0.2 |
| D1S235 | 1 | 233960389-233960673 | 2 | 163-199 | 3 | 0.159 | 6FAM-CAGCAAGAGTTCATGGGA | AACAGTCAATTACAAAATATGTGTG | 0.6 |
| D1S2630 | 1 | 165482761-165483032 | 2 | 264-272 | 3 | 0.170 | VIC-CCCAGAAGGTTGAGAGTGC | CAGTAATCCCATAGACAGTAAATCG | 0.4 |
| D4S403 | 4 | 13360002-13360189 | 2 | 213-237 | 3 | 0.179 | AGGTGGCCCTGAGTAGGAGT | 6FAM-TTTGAGGGAATGATTTGGGT | 0.4 |
| D10S1698 | 10 | 87895252-87895386 | 2 | 135-157 | 3 | 0.166 | PET-AGCTCTTTGCCCACTCCTG | GGGGAAGTTTGAACTGGGTC | 0.4 |
| D11S1321 | 11 | 75339935-75340147 | 2 | 197-215 | 3 | 0.174 | NED-AGCTGAGATCGCACCAT | TTCACTGCCATTTGTTGAC | 0.4 |
| D12S1723 | 12 | 130528689-130528851 | 2 | 157-175 | 3 | 0.251 | ATCCCGCCTCTGTAGAATG | NED-AAGGCCATGTGAGCATC | 0.2 |
| D14S1007 | 14 | 105049023-105049145 | 2 | 110-140 | 3 | 0.177 | AGCTCCTATATGTCTTCACACAG | VIC-CTCCATTCCCATACGTCC | 0.4 |
| D14S588 | 14 | 69290039-69290158 | 4 | 109-141 | 3 | 0.133 | GCCGAAAGAAAGAAAAAAGG | NED-CGAATGCATACTTGCTGTTG | 0.4 |
| D15S165 | 15 | 29047911-29048105 | 2 | 174-222 | 3 | 0.130 | GTTTACGCCTCATGGATTTA | PET-GGGCACACAGTCCCAA | 0.6 |
| D16S3401 | 16 | 171931-172078 | 2 | 151-189 | 3 | 0.144 | GAACAGGACATGGCTGTCAT | VIC-TCTACATGTGCCTAGAAGAC | 0.6 |
| D17S1799 | 17 | 50795628-50795719 | 2 | 78-96 | 3 | 0.177 | AGAAGGCCAGAGAGTAGATTTGTG | PET-TCTTAGAGCCTGTGTTGATAGTCG | 0.4 |

*NCBI build 36

†Forward primer is fluorescently labeled
