# Supplementary material for: CoAIMs: A Cost-Effective Panel of Ancestry Informative Markers for Determining Continental Origins
Source: PLoS One. 2010 Oct 15;5(10):e13443. doi: 10.1371/journal.pone.0013443 (PMC2955551; doi:10.1371/journal.pone.0013443)
Supplement: Table S4 — PCR conditions of the 3 multiplex PCRs for CoAIMs. (0.01 MB DOCX) [file pone.0013443.s007.docx]

|  | Panel 1 | Panel 2 | Panel 3 |
| --- | --- | --- | --- |
| Denature | 10 minutes at 94^o^C | 10 minutes at 94^o^C | 10 minutes at 94^o^C |
| Cycling (30 cycles) | 94^o^C for 45 seconds  60^o^C for 45 seconds  65^o^C for 45 seconds | 94^o^C for 45 seconds  56^o^C for 45 seconds  65^o^C for 45 seconds | 94^o^C for 45 seconds  60^o^C for 45 seconds  65^o^C for 45 seconds |
| Extension | 65^o^ for 10 minutes | 65^o^ for 10 minutes | 65^o^ for 10 minutes |
